# Targeting heat shock protein 90 with usnic acid relieves immune suppression via aryl hydrocarbon receptor-mediated mechanisms in lung cancer

**DOI:** 10.1186/s43556-025-00309-z

**Published:** 2025-10-15

**Authors:** Mücahit Varlı, Eun-Jung Ahn, Suresh R. Bhosle, Kyung-Sub Moon, Hyung-Ho Ha, Hangun Kim

**Affiliations:** 1 College of Pharmacy, Sunchon National University, 255 Jungang-Ro, Sunchon, Jeonnam 57922 Republic of Korea; 2 Department of Neurosurgery, Chonnam National University Hwasun Hospital and Medical School, 322 Seoyang-Ro, Hwasun, Jeonnam 58128 Republic of Korea

**Keywords:** Usnic acid, Heat shock protein 90, Aryl hydrocarbon receptor, Immune checkpoint molecules, Lung cancer

## Abstract

Immune evasion in lung cancer is closely associated with the dysregulation of molecular chaperones and immunoregulatory pathways. Heat shock protein 90 (HSP90), which is frequently overexpressed in lung cancer and correlates with poor prognosis, has emerged as a promising therapeutic target. Here, we investigated whether targeting the HSP90–aryl hydrocarbon receptor (AhR) axis with usnic acid (UA) could suppress immune evasion mechanisms in lung cancer. Through target prediction and molecular docking, UA-bead–based proteomic profiling, and in vitro assays, we identified HSP90 as a direct binding of UA. Unlike classical HSP90 inhibitors, UA downregulates HSP90 protein level and disrupts the HSP90–AhR complex, thereby promoting proteasomal degradation of AhR and reducing its half-life. This disruption suppresses AhR-associated gene expression and tryptophan metabolism-related markers under both AhR ligand-bound and ligand-free conditions. Additionally, the immune checkpoint molecules programmed death-ligand 1 (PD-L1) and inducible T-cell costimulator ligand (ICOSL) were markedly downregulated, demonstrating that UA modulates the tumor immune microenvironment via the HSP90–AhR axis. To assess its translational relevance, a water-soluble derivative of UA, potassium usnate (KU), was evaluated in a syngeneic lung cancer mouse model. KU treatment inhibited tumor growth in a dose-dependent manner, reduced tumor-associated macrophages and programmed cell death protein 1 (PD-1⁺) T cells, and increased the infiltration of cytotoxic T lymphocytes (CD8⁺) and helper T cells (CD4⁺). In addition, KU decreased the proliferation marker, antigen Kiel 67** (**Ki67⁺), and HSP90⁺ cell populations within tumors. Together, these findings demonstrate that UA/KU targets the HSP90–AhR axis, suppresses immune evasion pathways, and offers a novel immunomodulatory approach for lung cancer therapy.

**Supplementary Information:**

The online version contains supplementary material available at 10.1186/s43556-025-00309-z.

## Introduction

Lung cancer is the leading cause of cancer deaths worldwide, with a 5-year survival rate of 25% in the US (2013–2019) [1, 2]. Non-small cell lung cancer (NSCLC) makes up 85% of cases and is often diagnosed late. While smoking is the main risk factor, environmental and genetic factors also contribute [3, 4]. Given these limitations, increasing interest has focused on intracellular targets like heat shock protein 90 (HSP90), which plays a central role in supporting cancer cell survival and progression [5]. HSP90 interacts with client proteins and co-chaperones and plays an important role in many physiological processes and regulatory pathways, including apoptosis, cell cycle progression, cell viability, cell signaling, protein folding, and protein degradation [6, 7]. HSP90 controls the formation of tumours and several oncogenic processes, including angiogenesis, invasion, immune evasion, and apoptosis. Additionally, it stabilizes and activates various oncogenic markers that are linked to several pathways, including epidermal growth factor receptors (EGFR, ErbB2), protein kinase B (Akt), and hypoxia-inducible factor 1 alpha (HIF-1α) [8, 9].

HSP90 sustains a broad spectrum of oncogenic client proteins, including signaling molecules such as signal transducer and activator of transcription 3 (STAT3) and nuclear factor kappa B (NF-κB), thereby promoting major histocompatibility complex class I (MHC I) downregulation, PD-L1 upregulation, and overall immune checkpoint activation in tumors. These processes collectively impair immune recognition and effector T cell function [10, 11]. Independently, the aryl hydrocarbon receptor (AhR), which is stably retained in the cytoplasm by a dimer of HSP90 with the co-chaperones aryl hydrocarbon receptor-interacting protein (AIP) and p23, becomes activated upon ligand binding (e.g., kynurenine), translocates to the nucleus, and induces the expression of immunosuppressive genes, including those regulatory T cells and interleukins, while also upregulating immune checkpoints such as PD-1 and PD-L1 [12–15]. Together, the chaperoning function of HSP90 and the transcriptional activity of AhR establish a synergistic axis that amplifies immune suppression within the tumor microenvironment, highlighting their cooperative role in facilitating tumor immune evasion. However, the precise mechanisms by which the HSP90-AhR axis co-regulates immune escape pathways remain unclear, emphasizing the need to further investigate their direct and interdependent contributions to tumor-induced immune suppression.

Recent advances in our understanding of tumor heterogeneity, neoantigen expression, and the tumor microenvironment (TME) have laid the foundation for cancer immunotherapy, particularly in NSCLC [3, 16–18]. Immune checkpoint inhibitors (ICIs) that target PD-1/PD-L1 signaling have shown durable clinical responses in a subset of patients with lung cancer [15]. Other co-stimulators of T cells (ICOS, also known as the cluster of differentiation; CD278) and its ligand, ICOSL, are immunological checkpoints that are crucial for the formation of memory and effector T cells as well as some humoral immune responses, and several ICOS-targeting agents are currently under clinical evaluation for their therapeutic potential in cancer [19]. However, resistance and limited efficacy of ICIs highlight the need to explore additional immunoregulatory mechanisms. Notably, the expression of PD-L1 is regulated by intracellular signaling pathways, including those involving the AhR [15], a transcription factor stabilized by HSP90.

Lichen species that produce usnic acid (UA) have been used in traditional medicine worldwide. Numerous species of *Cladonia* are used in the treatment of pulmonary tuberculosis, and *Usnea* species are used for fever control and pain relief in Asia, Africa, and Europe. UA extracted from *U. barbata* is a component of contemporary medicinal and cosmetic formulations [20]. UA possesses anti-inflammatory, antiviral, antibacterial, antiosteoclastogenic, and anticancer properties [21]. In this regard, identifying the target-binding proteins and mechanisms of well-characterized natural compounds such as UA is critical for facilitating their clinical translation. Here, we identify HSP90 as a direct target of UA, demonstrating that UA disrupts the HSP90–AhR complex and restores antitumor immunity by reversing AhR-dependent immune suppression in NSCLC. Unlike conventional HSP90 inhibitors, whose clinical development has been limited by systemic toxicity, UA acts through a distinct mode of HSP90 modulation. Moreover, we evaluate potassium usnate (KU), a water-soluble UA salt with improved pharmacological properties [22], as a novel therapeutic candidate. To our knowledge, this is the first study to implicate KU in the suppression of lung cancer tumorigenesis and to reveal UA-mediated HSP90–AhR disruption as a previously unrecognized mechanism in lung cancer.

## Results

### Integrative network and experimental evidence identify HSP90 as a critical mediator of UA`s anticancer effects in lung adenocarcinoma

**Fig. 1:**
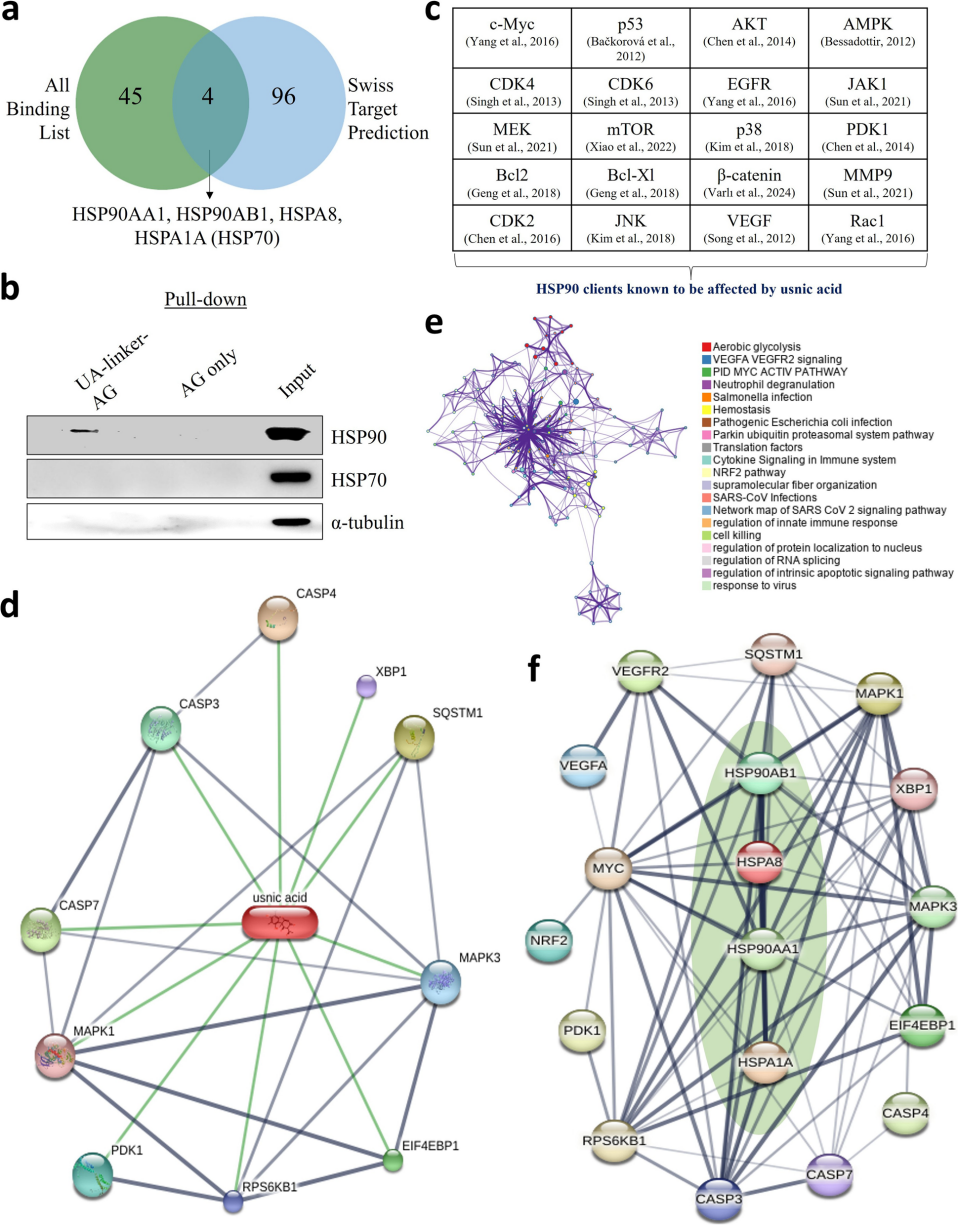
Analysis of HSP clients affected by usnic acid (UA). **a** Venn diagram showing the overlap between the target prediction list and UA direct binding list. **b** CaCo2 cell lysates were incubated with a chemical probe synthesized as described to pull down binding proteins. Proteins were identified by immunoblotting against HSP90 and HSP70 antibodies. **c** UA affects several HSP90 client proteins. **d** Drug–protein interaction network constructed by the STITCH database. **e** Network plot showing the results of pathway and process enrichment analyses using the UA direct binding protein list. **f** Interactions between HSPs and the indicated markers analyzed using the STRING database. AMPK: AMP-activated protein kinase; CDK: Cyclin-dependent kinases; IGF1R: Insulin-like growth factor 1 receptor; MTOR: Mechanistic target of rapamycin; PIK3CA: Phosphatidylinositol-4,5-bisphosphate 3-kinase catalytic subunit alpha; RAF1: RAF proto-oncogene serine/threonine-protein kinase; AKT1: AKT serine/threonine kinase 1; PDK1: 3-Phosphoinositide-dependent protein kinase-1; JAK1: Janus Kinase 1; JNK: c-Jun N-terminal Kinase; MEK: Mitogen-Activated Protein Kinase Kinase; MMP9: Matrix Metalloproteinase 9; BCL: B-cell lymphoma; Rac1: Ras-related C3 botulinum toxin substrate 1

**Fig. 2:**
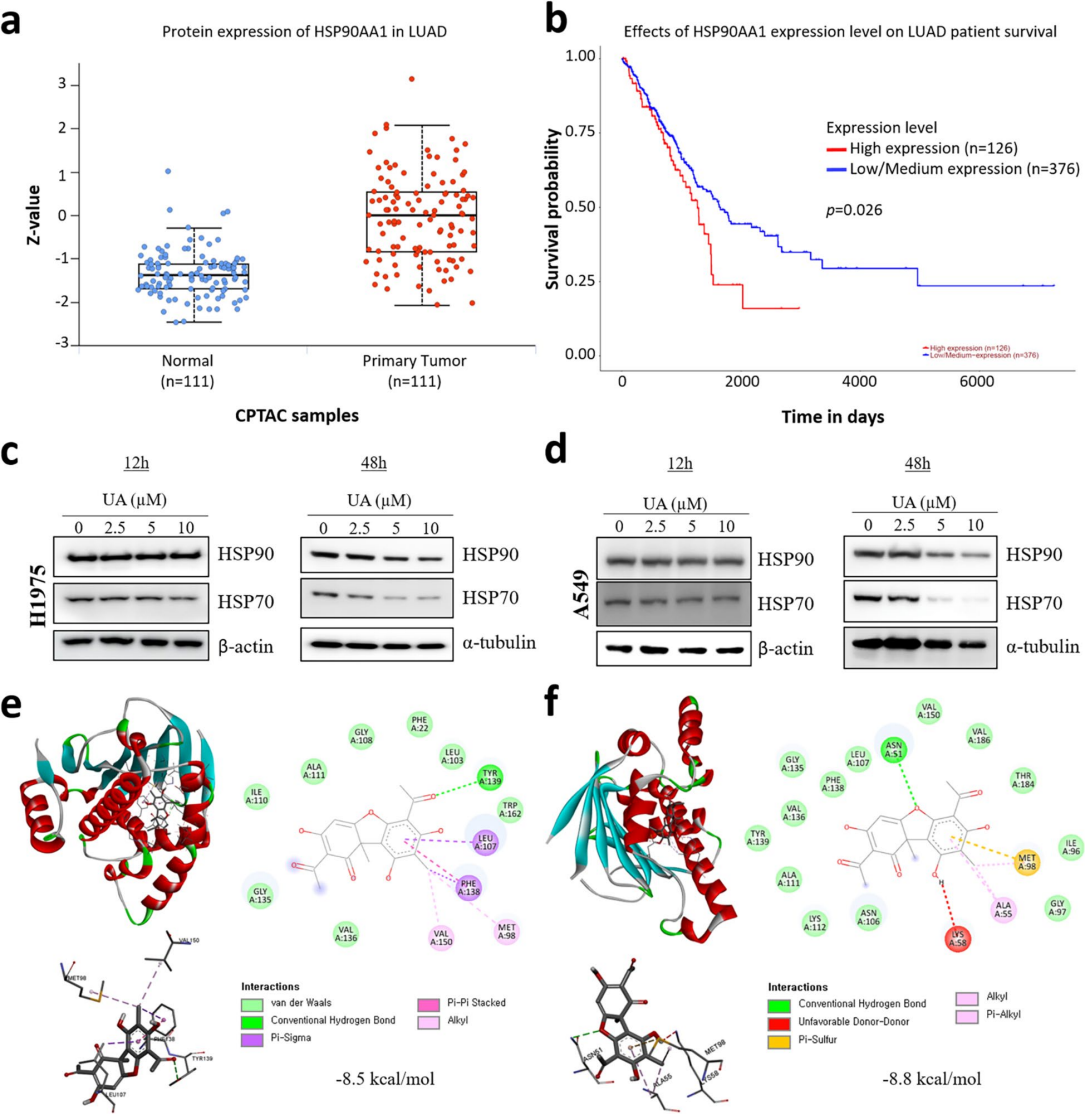
UA downregulates HSP90 and HSP70 protein expression in lung cancer cells. **a** Protein expression levels of HSP90AA1 in lung adenocarcinoma (LUAD) samples. Box plot shows standardized Z-scores of HSP90AA1 protein in normal tissues (n = 111) and primary tumor samples (n = 111) obtained from the CPTAC dataset via UALCAN (http://ualcan.path.uab.edu). **b** Prognostic impact of HSP90AA1 expression in LUAD patients. Survival analysis comparing high expression (n = 126) versus low/medium expression (n = 376) groups. A statistically significant difference in survival was observed (*p* = 0.026). **c**-**d** H1975 and A549 cells were seeded into 6-well plates, cultured overnight, and treated with various concentrations (2.5, 5, and 10 μM) of UA for 12 and 48 h. Cell lysates analyzed by SDS–PAGE and immunoblotting. **e**–**f** UA binding prediction to HSP90 (PDB: 1UY6, 3TUH). The predicted binding sites of HSP90 are indicated. HSP90 protein interactions (e.g., van der Waals interactions, hydrogen bonding) are shown in the BIOVIA Discovery Studio visualizer. Docking scores calculated by CB dock are shown in the figure

In a previous study, we developed a chemical probe to identify proteins that bind to UA in colorectal cancer [23]. We compared predicted targets (Table S1) with UA-binding proteins (Table S2) and identified HSPs as common hits (Fig. 1a). Pull-down assays using UA-linker-Affi-Gel beads showed that UA binds to HSP90 (Fig. 1b). UA is involved in the regulation of many HSP90 client proteins (Fig. 1c). A search of the STITCH database showed that UA interacts with caspases (CASP4, CASP3, CASP7), mitogen-activated protein kinases (MAPK1, MAPK3), pyruvate dehydrogenase kinase (PDK1), ribosomal protein S6 kinase B1 (RPS6KB1), eukaryotic translation initiation factor 4E-binding protein 1 (EIF4EBP1), X-box binding protein 1 (XBP1), and sequestosome 1 (SQSTM1) (Fig. 1d). Network clustering analysis based on UA-binding proteins indicated that the targets of UA are involved in aerobic glycolysis, apoptosis, immunity regulation, nuclear factor erythroid 2-related factor 2 (NRF2) pathway, vascular endothelial growth factor (VEGF) signaling, MYC pathway, and transcriptional regulators (Fig. 1e). We then investigated whether the data we obtained in Fig. 1d, e is in a correlation network with HSPs including HSP90 using the STRING database (Fig. 1f). Furthermore, HSP90AA1 is significantly overexpressed at the protein level in lung adenocarcinoma compared to normal tissue (Fig. 2a). In addition, elevated expression of HSP90 correlates with poor overall survival in lung cancer patients, underscoring its significance as a clinically relevant and potentially targetable molecular chaperone in lung cancer progression (Fig. 2b). The network analysis, direct target binding results, and clinical outcome results suggest that HSP90 may be the main regulator for UA on lung cancer. To examine the effect of UA on HSPs, A549 and H1975 cells were treated with 2.5, 5, and 10 μM UA for 12 and 48 h. Western blot analysis showed that UA downregulated HSP90 and HSP70 in a time- and dose-dependent manner (Fig. 2c, d). Next, we tested the binding of UA to HSP90 in silico using two sets of X-ray diffraction data for HSP90 from a protein database. The binding scores were −8.5 kcal/mol and −8.8 kcal/mol for two representative HSP90 protein structures, indicating strong predicted interactions (Fig. 2e, f). These results collectively confirm the functional and structural interaction between UA and HSP90.

### UA interacts with HSP90 and downregulates AhR by disrupting the HSP90-AhR interaction similar to the effect of the HSP90 inhibitor ganetespib (GAN)

**Fig. 3:**
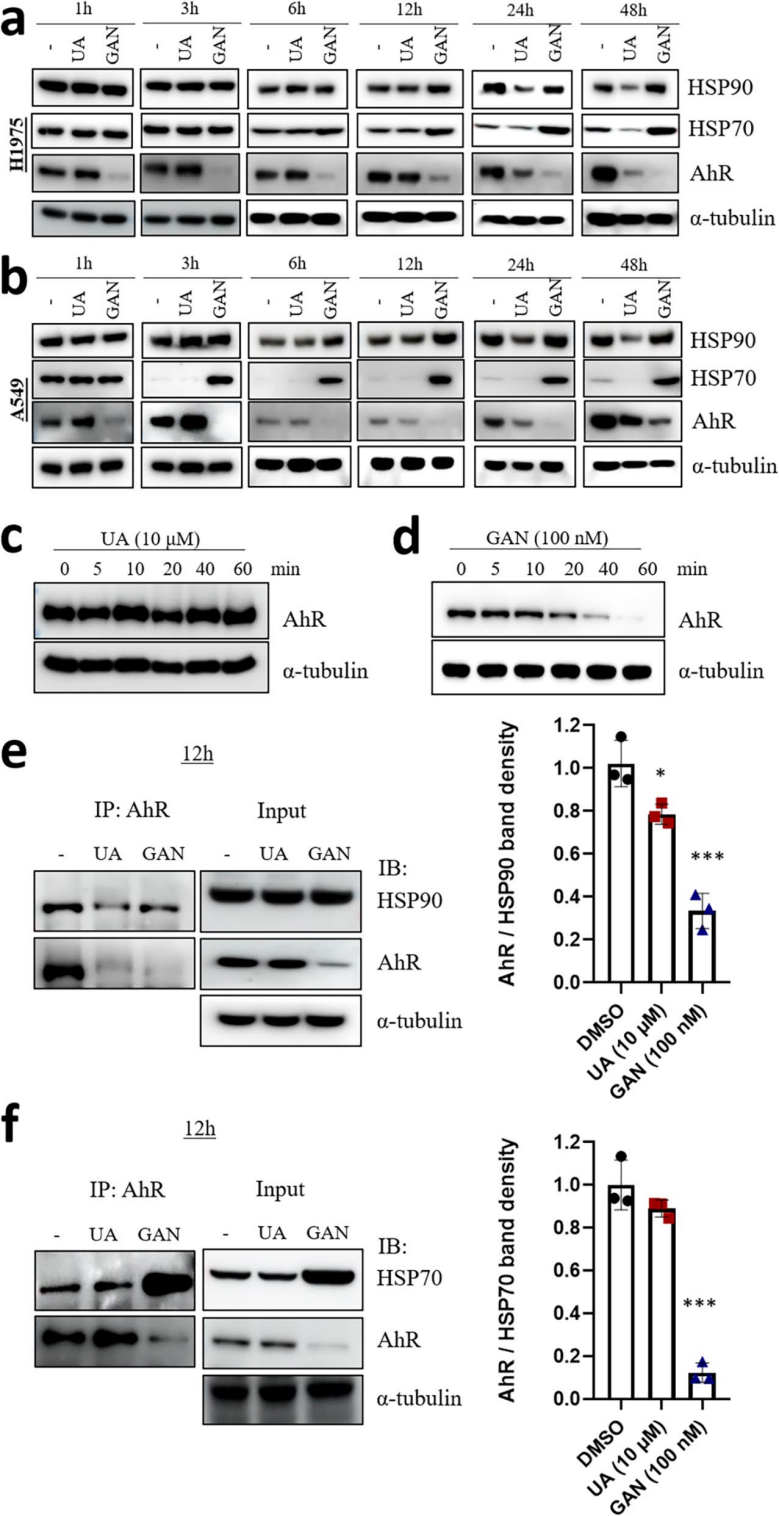
UA and GAN inhibit the AhR pathway via HSP90. **a** H1975 and **b** A549 cells were seeded into 6-well plates, cultured overnight, and treated with UA (10 μM) and GAN (100 nM) for 1–48 h. Cell lysates were subjected to SDS-PAGE followed by immunoblotting. **c**-**d** H1975 cells were treated with UA (10 μM) and GAN (100 nM) for 0–60 min, and AhR protein levels were detected by immunoblotting. **e**–**f** H1975 cells were treated with UA (10 μM) and GAN (100 nM) for 12 h, followed by immunoprecipitation with an AhR antibody and immunoblotting with HSP90, HSP70, and AhR antibodies. Whole-cell lysates (WCL) are shown in the figure. The relative levels of AhR IPed/HSP90 IPed or AhR IPed/HSP70 IPed relative to DMSO are given in the figure. Data are expressed as the mean ± SD, n = 3. * p < 0.05; ** p < 0.01; *** p < 0.001

HSP90 interacts with AhR to stabilize its conformation and ensure proper folding and function. HSP90 inhibitors such as geldanamycin and its derivatives bind to HSP90 at its ATP-binding site and inhibit its ATPase activity, thereby suppressing the chaperone function of HSP90 [24]. HSP90 inhibitors affect the stability of AhR by disrupting the HSP90-AhR complex, which alters the cellular localization and activity of AhR [25, 26]. To investigate UA’s mechanism of action, we compared it with the HSP90 inhibitor GAN (Fig. S1–3). H1975 and A549 cells were treated for 1–48 h, and HSP90, HSP70, and AhR levels were analyzed by western blot (Fig. 3a, b). UA reduced AhR after 12 h, whereas GAN did so within 1 h. In the first 60 min, UA showed no effect on AhR, while GAN markedly decreased its expression (Fig. 3c, d). To assess whether UA reduces AhR via enhanced degradation, cycloheximide assays were conducted. UA accelerated AhR decline compared to DMSO, indicating a shorter half-life. Co-treatment with the proteasome inhibitor MG132 restored AhR levels, suggesting UA promotes proteasome-dependent AhR degradation (Fig. S4). To furthermore, UA treatment led to a modest reduction in the interaction between AhR and HSP90, while its effect on the AhR–HSP70 interaction was negligible. In contrast, GAN, a classical HSP90 ATPase inhibitor, disrupted both AhR–HSP90 and AhR–HSP70 interactions markedly. These findings suggest that the effects of UA on AhR stability may be predominantly mediated through modulation of HSP90 expression levels. On the other hand, GAN-mediated disruption of AhR appears to be independent of HSP90 protein expression (Fig. 3e, f).

### Targeting HSP90 suppresses the induction of AhR-dependent gene expression

**Fig. 4:**
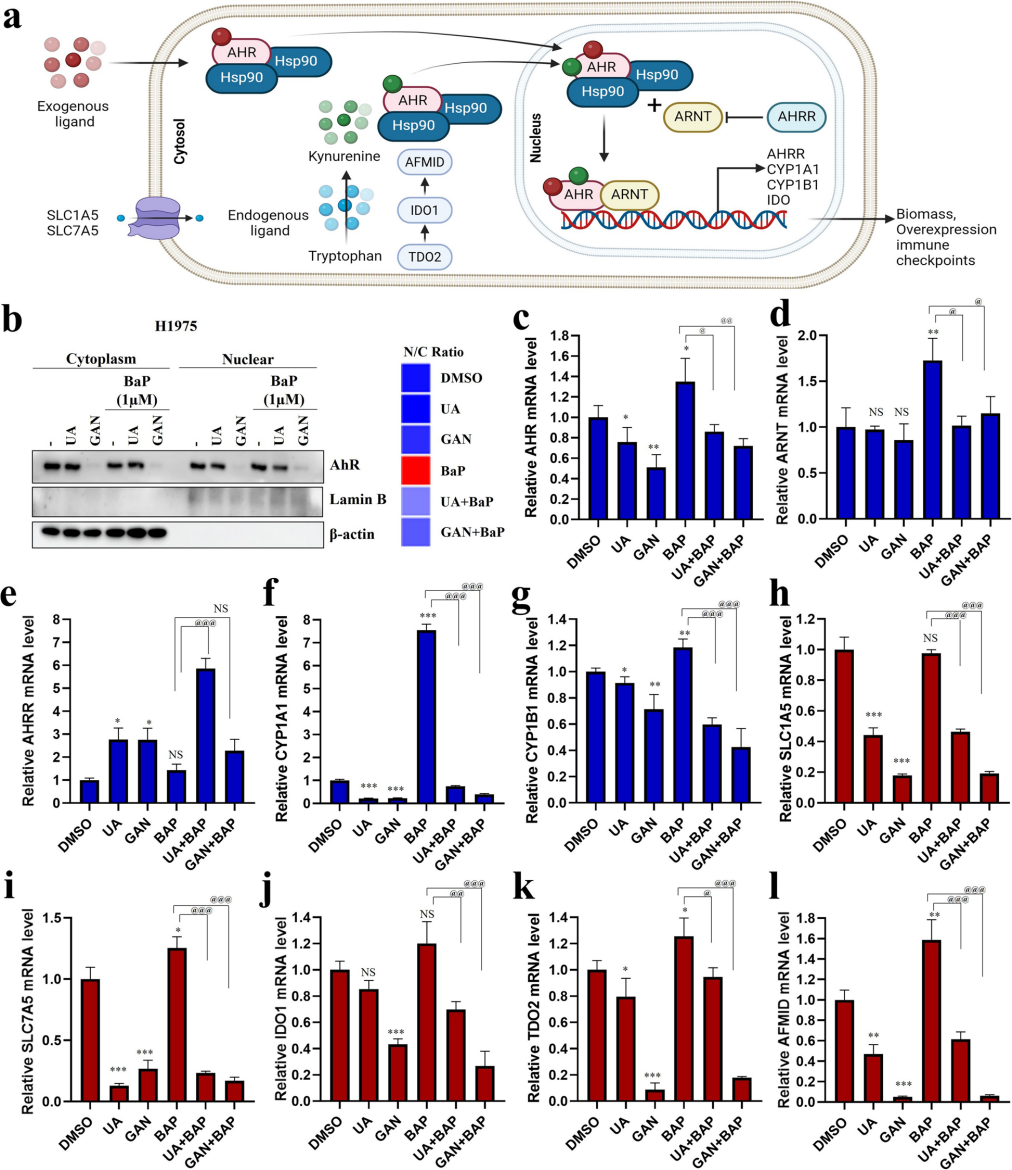
Inhibition of HSP90 decreases AhR-dependent gene expression. **a** Treatment with the HSP90-binding molecule UA and a known HSP90 inhibitor demonstrates the reversal of signaling in the activated AhR pathway using the ligand benzo[a]pyrene (BaP). The AhR-ARNT heterodimer binds to xenobiotic response elements (XREs) in the promoter region of target genes, initiating transcription. This increases the production of the natural ligand of AhR, kynurenine, promoting further activation of AhR through a feedback mechanism. **b** H1975 cells were treated with UA (10 μM) and GAN (100 nM) for 4 h prior to exposure to BaP (1 μM), and incubated for 1 h. Nuclear and cytoplasmic proteins were isolated, and AhR protein levels were detected by western blotting. **c**–**l** The mRNA expression of genes affected by AhR activation and markers related to tryptophan/kynurenine metabolism was also examined. H1975 cells were treated with UA (10 μM) and GAN (100 nM) for 4 h prior to exposure to BaP (1 μM) or DMSO, and incubated for 48 h. The mRNA levels of (**c**) AhR, (**d**) ARNT, (**e**) AhRR, (**f**) CYP1A1, (**g**) CYP1B1, (**h**) SLC1A5, (**i**) SLC7A5, (**j**) IDO1, (**k**) TDO2, and (**l**) AFMID were determined by qRT-PCR. *n* = 3; * p < 0.05; ** p < 0.01; *** p < 0.001; NS, no significant difference between DMSO vs treatment groups. ^@^ p < 0.05; ^@@^ p < 0.01; ^@@@^ p < 0.001; NS, no significant difference between benzo[a]pyrene (BaP) vs treatment groups

Disrupting AhR–HSP90 interaction impairs AhR ligand binding and nuclear translocation, which is blocked by HSP90 inhibitors such as those targeting dioxin-induced activation [27, 28]. We used the AhR ligand benzo[a]pyrene (BaP) to assess how UA and GAN affect AhR signaling. AhR-ARNT binds to XREs in target gene promoters, triggering transcription. This promotes a feedback loop via increased kynurenine production (Fig. 4a) [29–31]. Western blot showed that BaP-induced AhR nuclear translocation was suppressed in UA- and GAN-treated cells (Fig. 4b). Furthermore, we measured the mRNA levels of AhR, ARNT, aryl hydrocarbon receptor repressor (AhRR), cytochrome p450 family 1 subfamily members (CYP1A1, CYP1B1) in cells treated with or without BaP. Activated AhR, ARNT, CYP1A1, and CYP1B1 mRNA levels were downregulated by UA and GAN. However, AhRR mRNA levels suppressed by BaP treatment were reversed by UA and GAN treatment (Fig. 4c-g). Next, we investigated the mRNA expression levels of genes affected by AhR activation, as well as those of markers linked to tryptophan/kynurenine metabolism. Solute carrier family members (SLC1A5, SLC7A5), indoleamine 2,3-dioxygenase 1** (**IDO1), tryptophan 2,3-dioxygenase 2 (TDO2), and arylformamidase** (**AFMID) mRNA levels were downregulated by the UA or GAN treatment in the presence or absence of BaP (Fig. 4h-l). These findings emphasize the roles of HSP90-binding UA and HSP90 inhibitor GAN in regulating AhR pathways by modulating AhR nuclear translocation and gene activity, suggesting potential therapeutic strategies for managing genetic and metabolic effects of environmental toxins.

### Targeting HSP90 suppresses AhR ligand-activated immune checkpoint levels

**Fig. 5:**
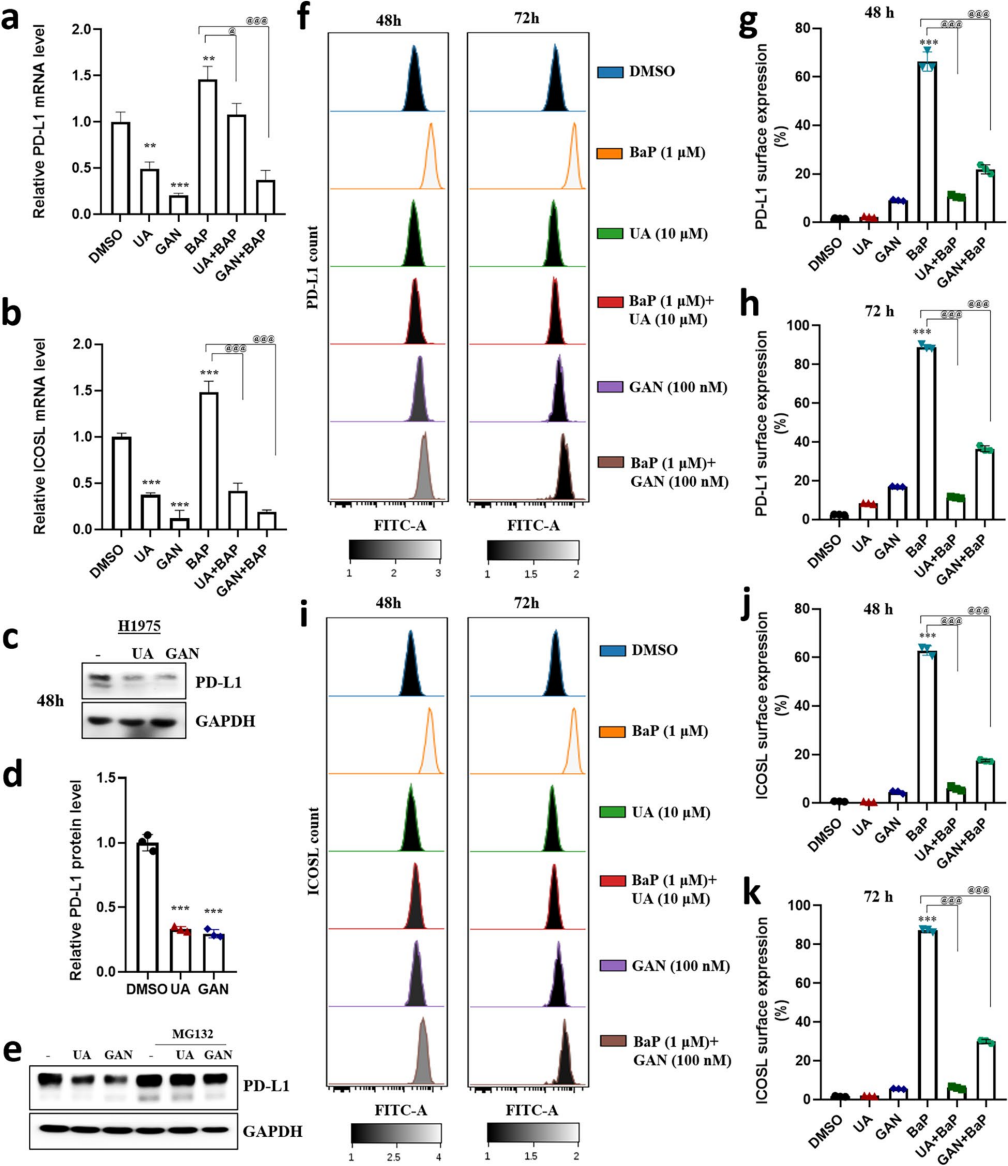
Inhibition of HSP90 reduces immune checkpoint levels activated by the AhR ligand. **a**-**b** H1975 cells were treated with UA (10 μM) and GAN (100 nM) for 4 h prior to exposure to BaP (1 μM), and incubated for 48 h. PD-L1 and ICOSL mRNA levels were determined by qRT-PCR. **c**-**d** H1975 cells were seeded into 6-well plates, cultured overnight, and treated with UA (10 μM) and GAN (100 nM) for 48 h. Cell lysates were subjected to immunoblotting for PD-L1. **e **Cells were treated with the proteasome inhibitor MG132 prior to treatment with vehicle control, UA, or GAN and subjected to western blotting to detect PD-L1 expression. GAPDH served as the loading control. **f **and** i** Cells were treated with DMSO, UA (10 μM), and GAN (100 nM) for 4 h prior to exposure to BaP (1 μM), and incubated for 48 and 72 h. Then, cells were analyzed by flow cytometry to determine surface expression of PD-L1 or ICOSL. **g**-**h** PD-L1 and (**j**-**k**) ICOSL surface protein expression were determined by flow cytometry. *n* = 3; * p < 0.05; ** p < 0.01; *** p < 0.001; NS, no significant difference between DMSO vs treatment groups. ^@^ p < 0.05; ^@@^ p < 0.01; ^@@@^ p < 0.001; NS, no significant difference between benzo[a]pyrene (BaP) vs treatment groups

The modulation of AhR in the TME suggests that AhR is a potential therapeutic target [32]. UA and GAN downregulated PD-L1 and ICOSL mRNA levels (Fig. 5a, b). UA and GAN downregulated the protein expression of PD-L1, and this effect was reversed by MG132 (Fig. 5c-e), suggesting that the effect of UA and GAN are mediated by the proteasomal degradation pathway. Flow cytometry experiments showed that the AhR ligand BaP increased the surface protein levels of PD-L1 and ICOSL, and this effect was reversed by UA and GAN (Fig. 5f–k). These results suggest that immune checkpoint proteins can be efficiently modulated by targeting HSP90 with UA and GAN via the AhR pathway. This suggests a possible therapeutic approach for boosting antitumor immunity.

### In silico assessment highlights pharmacokinetic and safety advantages of KU

In silico ADME predictions highlight several favorable properties of KU that may contribute to its improved bioavailability compared to UA. KU showed a notably higher Caco-2 permeability (log Papp: 1.235 vs. 0.781), suggesting enhanced passive absorption across intestinal epithelium. Furthermore, KU is not a substrate for P-glycoprotein, in contrast to UA, which is predicted to undergo efflux by this transporter. This distinction implies that KU may achieve higher intracellular and systemic concentrations by avoiding active efflux mechanisms that typically limit bioavailability. KU’s ability to inhibit P-glycoprotein I and its improved membrane permeability could enhance its effective absorption in vivo. Additionally, KU demonstrated a favorable predicted total clearance rate (log ml/min/kg = 2.729), indicating efficient systemic elimination, which may reduce toxicity risk while maintaining therapeutic plasma levels. Unlike UA, KU was not predicted to be hepatotoxic or AMES toxic, consistent with our previously in vivo and histopathological data [22]. Moreover, KU showed a higher predicted maximum tolerated dose in humans (log mg/kg/day = 0.923 vs. –0.411), and a higher oral LD_50_ in rats (2.729 vs. 1.778 mol/kg), indicating lower acute toxicity. In silico ADME-Tox analysis suggests that KU has better bioavailability, drug-like properties, and safety profile compared to UA, supporting its therapeutic potential (Table 1).

**Table 1:** Comparative in silico ADME prediction of KU and UA

Property	Model Name	Predicted value for KU	Predicted value for UA	Unit
Absorption	Water solubility	−4.593	−2.803	Numeric (log mol/L)
Absorption	Caco2 permeability	1.235	0.781	Numeric (log Papp in 10^–6^ cm/s)
Absorption	Intestinal absorption (human)	70.784	84.18	Numeric (% Absorbed)
Absorption	Skin Permeability	−2.809	−2.855	Numeric (log Kp)
Absorption	P-glycoprotein substrate	No	Yes	Categorical (Yes/No)
Absorption	P-glycoprotein I inhibitor	Yes	No	Categorical (Yes/No)
Absorption	P-glycoprotein II inhibitor	No	No	Categorical (Yes/No)
Distribution	VDss (human)	−0.022	0.358	Numeric (log L/kg)
Distribution	Fraction unbound (human)	0.248	0.437	Numeric (Fu)
Distribution	BBB permeability	−1.215	−0.533	Numeric (log BB)
Distribution	CNS permeability	−3.027	−3.086	Numeric (log PS)
Metabolism	CYP2D6 substrate	No	No	Categorical (Yes/No)
Metabolism	CYP3A4 substrate	Yes	No	Categorical (Yes/No)
Metabolism	CYP1A2 inhibitior	No	No	Categorical (Yes/No)
Metabolism	CYP2C19 inhibitior	No	No	Categorical (Yes/No)
Metabolism	CYP2C9 inhibitior	No	No	Categorical (Yes/No)
Metabolism	CYP2D6 inhibitior	No	No	Categorical (Yes/No)
Metabolism	CYP3A4 inhibitior	No	No	Categorical (Yes/No)
Excretion	Total Clearance	2.729	0.307	Numeric (log ml/min/kg)
Excretion	Renal OCT2 substrate	No	No	Categorical (Yes/No)
Toxicity	AMES toxicity	No	Yes	Categorical (Yes/No)
Toxicity	Max. tolerated dose (human)	0.923	−0.411	Numeric (log mg/kg/day)
Toxicity	hERG I inhibitor	No	No	Categorical (Yes/No)
Toxicity	hERG II inhibitor	No	No	Categorical (Yes/No)
Toxicity	Oral Rat Acute Toxicity (LD_50_)	2.729	1.778	Numeric (mol/kg)
Toxicity	Oral Rat Chronic Toxicity (LOAEL)	1.375	2.303	Numeric (log mg/kg_bw/day)
Toxicity	Hepatotoxicity	No	Yes	Categorical (Yes/No)
Toxicity	Skin Sensitisation	No	No	Categorical (Yes/No)
Toxicity	*T.Pyriformis* toxicity	0.295	0.358	Numeric (log ug/L)
Toxicity	Minnow toxicity	−1.367	2.263	Numeric (log mM)

log Papp: Permeability Apparent; VDss: Volume of Distribution at steady state; BBB: Blood–Brain Barrier; CNS: Central Nervous System; CYP2D6, CYP3A4, CYP1A2, CYP2C19, CYP2C9: Cytochrome P450 ezymes; OCT2: Organic Cation Transporter 2; hERG I inhibitor/hERG II inhibitor: Human Ether-a-go-go-Related Genes; LD_50_: Lethal Dose 50%; LOAEL: Lowest Observed Adverse Effect Level

### KU, a water-soluble of HSP90-binding UA salt, suppresses tumorigenesis in a murine lung cancer model

**Fig. 6:**
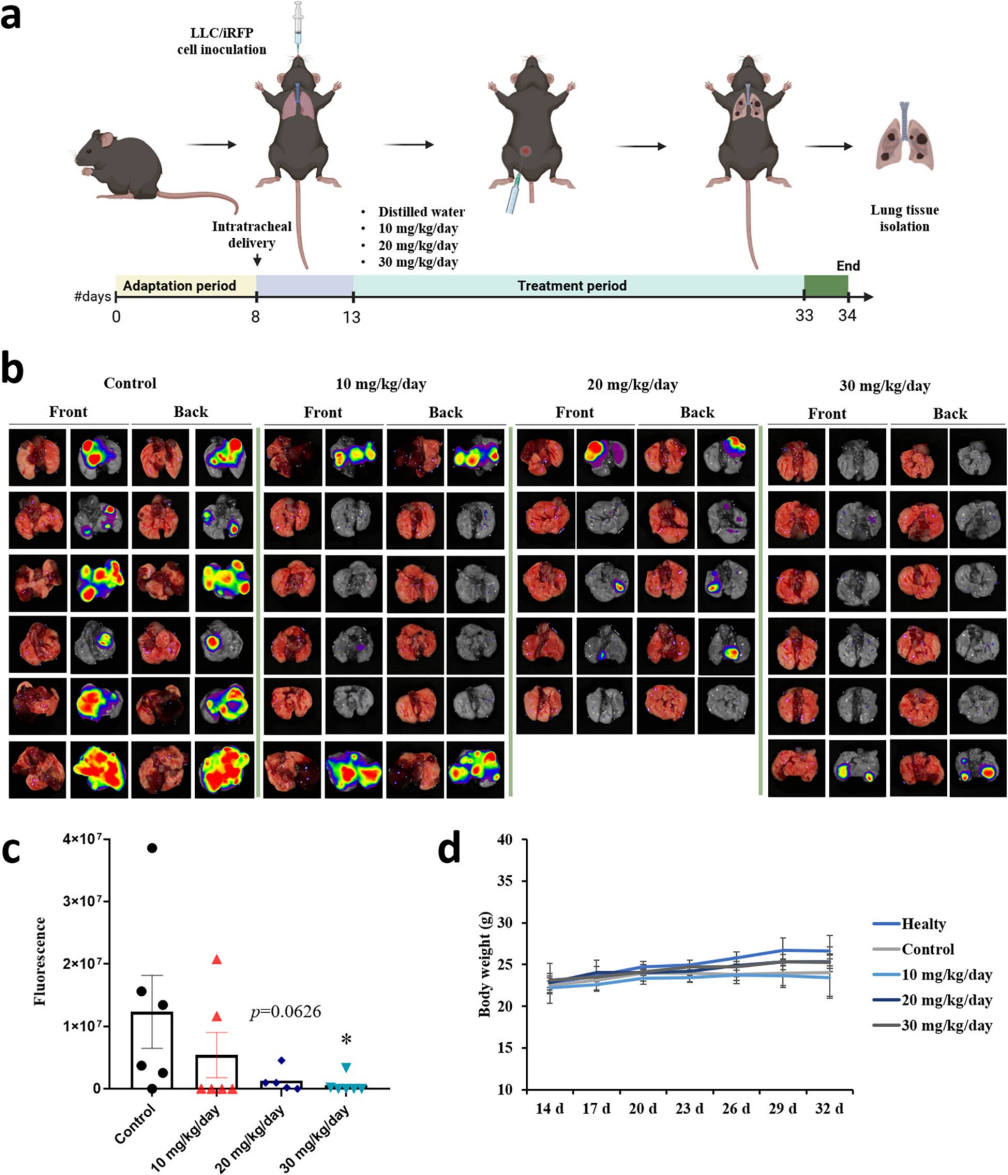
KU, a water-soluble UA salt, suppresses tumorigenesis in a murine lung cancer model. **a** Schematic of the in vivo experimental procedure. A total of 4 × 10^6^ LLC/iRFP cells were inoculated via the trachea. After 5 days, mice received intraperitoneal administration of distilled water as the control (n = 6), 10 mg/kg/day KU (n = 6), 20 mg/kg/day KU (n = 5), or 30 mg/kg/day KU (n = 6) daily for 13–33 days. **b**-**c** To evaluate tumorigenesis, the total fluorescence emitted from lung expressing iRFP was measured and counted. An asterisk indicates a significant difference between the indicated groups * p < 0.05. **d** Body weight of healthy, control, and 10, 20, and 30 mg/kg/day KU groups

As an initial step before proceeding with the in vivo murine lung cancer model, we confirmed KU’s activity in murine lung carcinoma, LLC, cells by observing reduced expression of HSP90 and PD-L1 (Fig. S5). We evaluated the therapeutic efficacy of KU by establishing lung tumors derived from LLC/near-infrared fluorescent protein (iRFP) cells via intratracheal transplantation in C57BL/6 mice. Figure 6a shows a schematic representation of the experimental procedure. KU was administered intraperitoneally at 10, 20, and 30 mg/kg/day. Lung tumorigenesis was evaluated by detecting total fluorescence emitted from tumor nodules expressing iRFP. Tumorigenesis was suppressed by KU in a dose-dependent manner (Fig. 6b, c). KU at 30 mg/kg/day significantly reduced tumorigenesis compared with the control group and did not show toxic effects (Fig. 6d). These results suggest that KU exerts strong anticancer effects without causing toxicity, indicating that it may be used as a lung cancer treatment.

### Effects of KU, a water-soluble of HSP90-binding UA salt, regulates tumor immune landscape by decreasing TAMs and PD-1 + exhausted T cells while modulating CD4 + populations

**Fig. 7:**
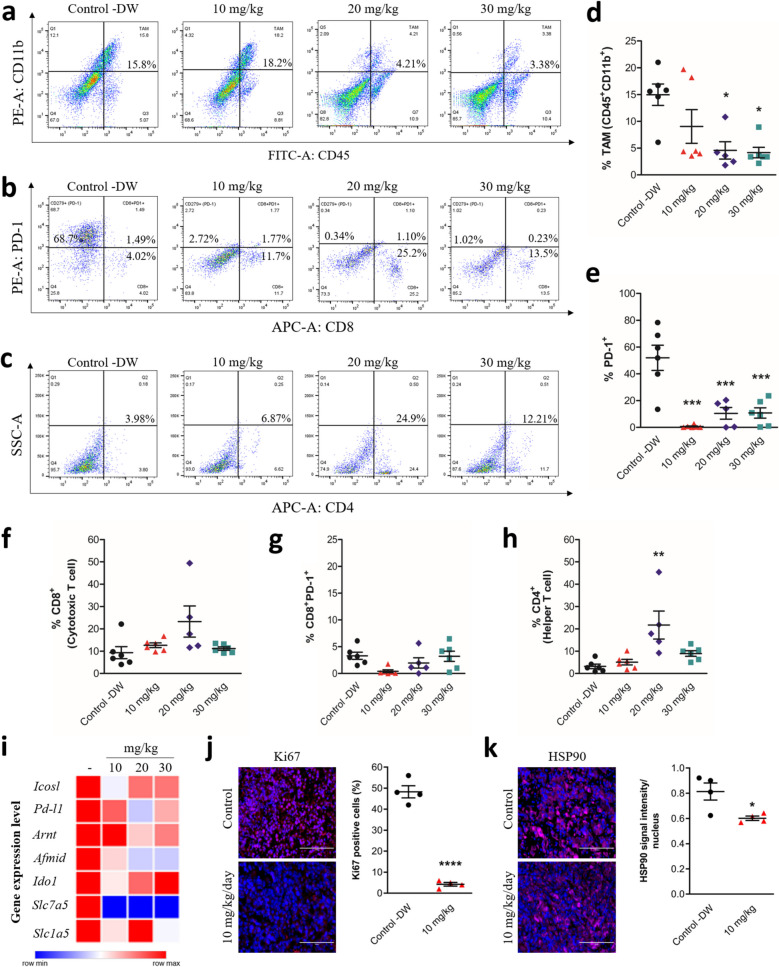
KU modulates tumor immune microenvironment by reducing TAMs and PD-1 + cells while altering CD4 + populations and proliferation markers by targeting HSP90. Flow cytometric analysis of immune cell populations in the TME following treatment with different doses (10, 20, and 30 mg/kg) of KU. **a** Representative flow cytometry plots showing the percentage of TAMs (CD45 + CD11b +) after treatment with different concentrations of KU. **b** Flow cytometry plots illustrating the distribution of CD8 + PD-1 + (exhausted cytotoxic T cells) in the TME. **c** Flow cytometry plots illustrating the distribution of CD4 + (T-helper cells) in the TME. **d** Quantitative analysis of TAMs (CD45 + CD11b +) in different treatment groups shows a dose-dependent decrease in TAM populations with increasing doses of KU. **e** Quantification of PD-1 + cells indicates a significant reduction in PD-1 expression with increasing doses of KU. **f** Analysis of CD8 + cytotoxic T cells in the CD8 + population in KU-treated groups. **g** Analysis of the percentages of CD8 + PD-1 + cells (exhausted cytotoxic T cells) shows a slight increase in the higher dose groups. **h** Quantification of CD4 + helper T cells shows a significant increase in the 20 mg/kg treatment group. Significant differences compared with the control group are indicated by *p < 0.05, **p < 0.01, and ***p < 0.001. Group sizes were as follows: control, n = 6; 10 mg/kg/day KU, n = 6; 20 mg/kg/day KU, n = 5; 30 mg/kg/day KU, n = 6. **i** Heatmap showing relative mRNA expression levels of selected immune checkpoint regulators (*PD-L1, ICOSL*), AhR target gene *ARNT*, and metabolic markers (*AFMID, IDO1, SLC7A5, SLC1A5*) in lung tissues from control and KU-treated mice (10, 20 and 30 mg/kg). Gene expressions were assessed by qRT-PCR and normalized to housekeeping genes. **j** Representative immunofluorescence images of lung tumor sections from control and KU-treated mice (10 mg/kg) stained for Ki67 (a proliferation marker, red) and DAPI (nuclei, blue), n = 4, ****p < 0.0001. Scale bar = 100 μm. **k** Representative immunofluorescence images of lung tumor sections from control and KU-treated mice (10 mg/kg) stained for HSP90 (red) and DAPI (nuclei, blue), n = 4, *p < 0.05. Scale bar = 100 μm

To further characterize the effect of KU on cancer immunity, we measured CD11b +, CD8 +, CD4 +, and PD-1 + cell populations in isolated lung tissues by flow cytometry. KU treatment decreased the levels of TAMs in a dose-dependent manner, as defined by CD45 + CD11b + populations. Treatment with KU at doses of 10, 20, and 30 mg/kg changed the percentage of TAMs from 15.8% in the control group to 18.2%, 4.21%, and 3.38%, respectively (Fig. 7a, d), indicating a threefold decrease in TAMs at the highest dose of 30 mg/kg. This suggests that KU is effective against such pro-tumorigenic cells that promote immune evasion. Notably, CD11b is a marker shared by multiple innate immune cell subsets, including dendritic cells (DCs) and certain NK cell populations, particularly under inflammatory conditions [33–36]. Therefore, the observed reduction in the CD11b + may not be limited to TAMs but could also indicate a broader remodeling of the innate immune landscape, potentially affecting both antigen-presenting and cytotoxic cell functions within the TME. KU treatment also decreased the levels of PD-1 + T cells (Fig. 7b), or exhausted T cells. The levels of PD-1 + cells decreased from 70.19% to 1.25% in response to 30 mg/kg KU, indicating a significant for all dose suppression (p < 0.001). This suggests a potential reversal of immune suppression in the TME upon KU treatment (Fig. 7e). No significant changes were observed in CD8 + T-cell populations in relation to TAMs or PD-1 + cells, suggesting that the antitumor activity of KU does not depend primarily on the activation of CD8 + T cells, and may be mediated by other immune-modulating processes within the TME (Fig. 7e-g). PD-1⁺ expression is not only a hallmark of exhausted effector T cells but is also associated with regulatory T cells (Tregs), which can express PD-1 to enhance their suppressive functions within the TME [37]. Therefore, the observed reduction in PD-1⁺ cells after KU treatment may reflect a decrease in both exhausted effector T cells and immunosuppressive Tregs, contributing to the restoration of effective anti-tumor immunity. The dynamics of the CD4 + population may offer insight into shifts in both helper and regulatory T-cell activity, reflecting broader changes in the immune regulatory landscape within the TME [38]. By contrast, the percentage of CD4 + helper T cells significantly increased in the 20 mg/kg treatment group compared with the control (p < 0.01), suggesting the specific induction of helper T-cell responses by this intermediate dose. At the high dose of 30 mg/kg, however, the levels of CD4 + cells returned to near-control values, indicating a dose-dependent response (Fig. 7c, h). Given that Tregs represent a subset of the CD4 + population, this transient increase may also reflect a shift in the balance between immunostimulatory and immunosuppressive CD4 + subsets. To further investigate the molecular impact of KU, we analyzed the mRNA expression profiles of several markers such as AhR target gene ARNT, metabolic markers and immune checkpoints in lung tissues. As shown in the heatmap (Fig. 7i), KU treatment led to the downregulation of PD-L1, ICOSL, ARNT, IDO1, AFMID, SLC1A5, SLC7A5. Finally, immunohistochemistry revealed a significant reduction in Ki67–positive cells (Fig. 7j) in KU-treated tumor tissues, reflecting a clear decrease in proliferative activity and the expression of HSP90, target molecule, was markedly downregulated following KU treatment (Fig. 7k). Taken together, these findings indicate that KU may increase the efficacy of immunotherapy by targeting HSP90`s mechanisms in the lung cancer.

## Discussion

UA is a secondary metabolite of lichens with a wide range of biological activities. It possesses antibacterial, anti-inflammatory, antiviral, antioxidant, and antitumor activities. The biological effects of UA are under investigation in various disease models by assessing how the compound targets different cellular mechanisms. UA acts on a wide range of cancer cells, arresting cell cycle progression and inducing apoptosis. UA can inhibit the growth of tumor cells and induce programmed cell death (apoptosis) in different types of cancer cells. This mode of action enables the evaluation of UA for its anticancer potential [21, 23, 39–48]. In this study, we aimed to elucidate the molecular mechanisms underlying the effects of UA by characterizing its targeting of HSPs using in silico and in vitro methods. The findings of the current study can be summarized as follows: (1) UA binds to HSP90, which downregulates HSP90 and HSP70 protein expression in lung cancer cells; (2) UA decreases AhR stability by disrupting the HSP90–AhR complex, thereby perturbing AhR-regulated pathways; (3) UA and GAN inhibit AhR nuclear translocation and gene expression in the presence of BaP, thereby AhR related genes and tryptophan pathway downregulated; (4) UA and GAN decrease PD-L1 and ICOSL levels via the AhR signaling pathway, which may enhance antitumor immunity; (5) KU, a bioavailable form of UA, suppresses lung tumor growth; and (6) KU decreases TAMs and PD-1 + T cells, suggesting an anti-immune evasion effect, and selectively increases CD4 + T-cell populations; (7) KU treatment downregulated mRNA expression of key immune and metabolic markers and significantly reduced Ki67 and HSP90 protein levels in ex vivo analysis.

HSP90 stabilizes numerous proteins with a wide range of biological functions. Under normal conditions, HSP90 is expressed in most human tissues and is essential for preserving cellular proteostasis [6]. However, in cancer, the HSP90 chaperone is hijacked to support oncogenesis and tumor progression through the stabilization of mutated proteins, the induction of dysregulated signaling pathways driven by oncogenes, and by promoting rapid proliferation of cells and tumor growth [49]. The preclinical evaluation of HSP90 inhibitors is an active area of interest in cancer research because of the potential of these inhibitors to destabilize multiple oncogenic signaling pathways simultaneously [50]. The first inhibitors developed were natural products, such as geldanamycin and radicicol [51]; these compounds showed great promise in preclinical studies because of their ability to kill cancer cells through the pharmacological destabilization of essential signaling pathways. Despite the development of second- and third-generation HSP90 inhibitors with improved drug-like properties, none of the 19 candidates has gained FDA approval due to limited and short-lived clinical responses [52]. For instance, the first-generation geldanamycin (GA) derivative IPI-504 showed only a 26% ORR in a phase I trial in advanced NSCLC patients when combined with docetaxel [53]. Another phase I trial tested SNX-5422 combined with carboplatin and paclitaxel in patients with advanced lung cancer, establishing a maximum tolerated dose (MTD) of 100 mg/m^2^. Among the 18 response-evaluable NSCLC patients, 33% (6/18) achieved a partial response, 56% (10/18) had stable disease, and 11% (2/18) experienced disease progression [54]. The limited clinical success of HSP90 inhibitors is due to toxicity, dosing challenges, and resistance. Ongoing efforts aim to enhance their efficacy through combination therapies and improved trial strategies [52, 55, 56]. Therefore, the discovery of new HSP90-targeting compounds may be a promising approach to overcome current difficulties.

HSP90 inhibitors induce endoplasmic reticulum (ER) stress, leading to impaired proteostasis in cancer cells and consequently activating unfolded protein response (UPR) pathways. This process induces apoptosis by causing an increase in proapoptotic molecules (e.g. CHOP) and activation of JNK and intrinsic caspase pathways. HSP90 inhibitors also trigger the intracellular heat shock response (HSR) [57]. This response leads to increased expression of other chaperone proteins such as HSP70 and HSP27, which may promote the survival of cancer cells. This may limit the therapeutic efficacy of HSP90 inhibitors [58]. GAN (STA-9090), a classical HSP90 inhibitor, upregulated HSP90 and HSP70 in A549 and H1975 cells, consistent with resistance via heat shock response (HSR). In contrast, UA downregulated these proteins and showed weaker cytotoxicity in non-cancerous Beas-2B cells compared to GAN, indicating a distinct mechanism and potentially better safety. UA may reduce resistance development by limiting chaperone expression while being less harmful to healthy cells. UA emerges as a promising alternative to classical inhibitors, showing not only cytotoxicity but also suppressing cell motility and metastasis at low concentrations. Its reported inhibition of epithelial-mesenchymal transition (EMT) suggests UA/KU targets tumor cell death as well as invasion and metastasis, highlighting its potential as a versatile anti-metastatic agent [22, 48, 59, 60].

Despite differing effects on HSP expression, UA and GAN both converge on reducing AhR levels through disruption of HSP90-mediated stabilization, leading to degradation. This shared mechanism reflects the loss of chaperone support. Notably, AhR ligands include BaP, dioxin, and polycyclic aromatic hydrocarbons [61]. These compounds can enter the body through industrial by products, cigarette smoke and air pollution. Environmental AhR ligands can increase inflammation, which can trigger cancer development by suppressing the immune system. In addition, AhR induces the expression of xenobiotic-metabolizing enzymes, which are members of the cytochrome P450 family of drug-metabolizing enzymes that includes CYP1A1, CYP1A2, and CYP1B1 following nuclear transfer from the AhR-HSP90-p23-XAP2 complex [62]. The HSP90-targeting compounds UA and GAN downregulated AhR, its target genes CYP1A1 and CYP1B1, and the immune checkpoints PD-L1 and ICOSL involved in immune evasion.

AhR is a nuclear receptor that mediates the response of cells to environmental toxins, but it also plays a major role in the regulation of the immune system through L-Kyn and other endogenous ligands [63]. AhR signaling increases the expression of exhaustion markers such as PD-L1, 4-1BBL, GAL-9, HVEM, ICOSL, LAG-3, and TIM-3, leading to the loss of effective antigen responsiveness in T cells [64, 65]. AhR activation can alter the function of immune cells (especially T cells) and promote the development of an immunosuppressive microenvironment and cancer or other disease therapies directed towards inhibition of AhR or IDO/TDO enzymes are demonstrated [66, 67]. Moreover, both innate and adaptive immune cells are modulated by the AhR, which plays a complicated and context-dependent role in tumor immunity. Macrophage polarization toward an immunosuppressive M2-like phenotype is influenced by AhR activation, which also promotes NK cell cytotoxicity and dendritic cell differentiation in both pro- and anti-tumor orientations. AhR stimulates tissue-resident memory-like cells associated with enhanced anti-tumor responses in CD8 + T cells while simultaneously driving T cell exhaustion. Likewise, AhR controls CD4 + T cell subsets, giving rise to regulatory T cells (Tregs) and depending on the situation, these cells can either promote or inhibit the growth of tumors [32, 68–77]. Selective AhR inhibition combined with PD-1 blockade increases the efficacy of anti-PD-1 therapy and slows the progression of tumors overexpressing IDO or TDO [78]. Treatment with an AhR inhibitor increases the expression of CD4 + T cells in a dose-dependent manner [15]. TAMs, which often exhibit immunosuppressive phenotypes that support tumor progression, are key players in the Kyn-AhR pathway of immunosuppression [73]. In this study, we demonstrated that KU has in vivo activity in lung cancer by suppressing tumorigenesis, downregulating TAMs (CD11b) and PD-1 + cell populations and inducing CD4 + cell populations.

Our recent study, we demonstrated that UA directly binds to 14–3-3 proteins and suppresses colorectal cancer progression [23]. In the current study, we systematically report all direct protein targets of UA and, using SwissTargetPrediction as a ligand-based computational tool, we identified HSP90 as a key molecular target, supported by both in silico predictions and experimental validation. While the clinical application of UA is limited due to its poor solubility and reports of liver toxicity as a dietary supplement [60, 79, 80], the form of UA we used in our study, KU, demonstrating improved bioavailability and favorable safety profiles, is a more suitable candidate for clinical applications. KU has demonstrated potential antitumor effects in vivo across various models, including colorectal cancer liver metastasis [22, 60], orthotopic breast cancer [81], orthotopic glioblastoma [82], and, in the present study, a lung cancer model. Our previous study supports KU’s safety, showing no hepatotoxicity up to 20 mg/kg in mice, with stable ALT/AST levels and normal liver histology. KU-treated mice had lower AST levels and reduced tumor mitotic activity (decreased pHH3 staining), indicating antitumor effects [22]. In silico analysis predicts KU has better membrane permeability, lower efflux, higher clearance, and reduced toxicity compared to UA, suggesting improved pharmacological properties and a safer profile warranting further study. The multifaceted effects of KU, such as HSP90 inhibition, suppression of AhR pathways, reduction of immune checkpoints and reorganization of the tumor microenvironment, make it a potent molecule that should be evaluated in the clinic both as a direct tumor suppressor and as an immune system modulator.

This study has limitations. In vivo experiments were limited to a single tumor model, and KU’s efficacy was not assessed in combination with standard therapies or for long-term toxicity. Mechanistic insights were based on target inference without genetic loss-of-function models. Future work should include multiple tumor models, extended pharmacokinetic profiling, and functional assays to better define the therapeutic potential of UA/KU.

In conclusion, UA targets HSP90 to destabilize AhR and block downstream signaling, thereby modulating the immune system in lung cancer and the tumor microenvironment. By regulating immune checkpoint proteins such as PD-L1 and ICOSL, UA may enhance antitumor immunity. KU, a highly bioavailable UA derivative, inhibited lung tumor growth by altering the expression of TAMs, PD-1⁺ T cells, and CD4⁺ T cells.

## Materials and methods

### Cell culture and reagents

Cell lines used in this study have undergone STR analysis, which was performed by the Korean Cell Line Bank (KCLB, Seoul, Republic of Korea) and were confirmed to be mycoplasma-free. Lung cancer cells A549, H1975, LLC-iRFP and human lung epithelial BEAS-2B cells line were used in this research. All cells were incubated in 5% CO_2_ in a humidified atmosphere at 37 °C. All cells were cultured in RPMI/DMEM supplemented with 10% fetal bovine serum and 1% penicillin–streptomycin solution. UA was purchased from Sigma-Aldrich. GAN (STA-9090) was purchased from Selleckchem. KU was prepared as previously described [22, 83]**.** MG132 and cycloheximide were purchased from Sigma-Aldrich.

### Cytotoxicity assay by MTT

Cells were used in the colorimetric quantification of MTT (3-(4, 5-dimethylthiazol-2-yl)−2, 5-diphenyltetrazolium bromide) test to determine the viability of the cells. In summary, 96-well plates were seeded with 3 × 10^3^ cells/well, allowed to grow overnight, and then incubated with drugs at the stated doses (100–0.0487 µM) for 48 h. This concentration range has also been used in previous studies conducted on lung cell lines [44, 84–86]. Cells were lysed with DMSO following a 4-h incubation period with MTT solution. A microplate reader was used to measure the absorbance value at 570 nm, and Gen 5 (2.03.1; BioTek, VT, USA) was used for analysis.

### Western blotting

To prepare whole cell lysates, cells were plated into 6-well plates and left to incubate for the entire night. Following the recommended incubation period, cells were lysed using lysis buffer. Protein concentrations were determined using the bicinchoninic acid (BCA) assay (Thermo Fisher Scientific, Waltham, MA, USA). Horseradish peroxidase-conjugated secondary antibodies (Thermo Fisher Scientific) were used in conjunction with luminescence imaging and the Immobilon Western Chemiluminescent HRP Substrate Kit (Millipore, Billerica, MA, USA) to detect antibodies (Table S3).

### Pull down assay

In pulldown buffer (50 mM HEPES, 30 mM NaCl, 1 mM EDTA, 2.5 mM EGTA, 0.1% Tween-20, cocktail inhibitor, pH 7.5), UA beads were incubated with CaCo2 cell lysates for 12 h at 4 °C. Following a thorough wash with wash buffer (pulldown buffer), bead-bound proteins were separated on SDS-PAGE and subjected to immunoblot analysis employing antibodies against candidate proteins.

### Immunoprecipitation

H1975 cell lysates were immunoprecipitated overnight at 4 °C with primary antibody, followed by 3 h with Protein A/G Sepharose (Thermo Scientific, 20422). After six washes with lysis buffer, the bound proteins were separated by SDS-PAGE and analyzed by immunoblotting.

### Subcellular fraction

A subcellular protein fractionation procedure was used to create the cytoplasmic and nucleus fractions in accordance with the guidelines provided by Thermo Scientific (NE-PERTM Nuclear and Cytoplasmic Extraction Reagents, catalog number: 78833). Probing for β-actin in the cytoplasm and Lamin B in the nucleus was used to determine the fraction purity.

### Immune checkpoints surface protein expression

Surface protein expression was analyzed in H1975 cells, which were plated at a density of 2 × 10^5^ cells/well in a 6-well plate and incubated overnight. The cells were then treated with DMSO or compounds 4 h before exposure to BaP (1 µM) and incubated for 48 or 72 h. Cell pellets were stained with anti-PD-L1 (Cell Signaling, #13,684) or anti-ICOSL (Invitrogen, #16–5859-82) antibodies in 100 μL aliquots (1 × 10⁶ cells) at 4 °C for 30 min in the dark after the cells had been washed three times. The cells were treated with the secondary antibody anti-Rabbit IgG (H + L) (Cell Signaling, Alexa Fluor 488 conjugate, #4412) at 4 °C for 30 min in the dark after being washed twice with FACS buffer. Following further washing, a Cytoflex flow cytometer (Beckman Coulter Life Sciences, Indianapolis, IN, USA) and CytExpert 2.0.0.152 software were used to evaluate the labeled cells using flow cytometry.

### Protein–ligand interaction modeling

The crystal structures of the HSP90 protein, retrieved from the PDB database (PDB IDs: 1UY6, 3TUH), were used to examine the interaction mechanism between the two protein targets and their ligand binding sites. Molecular docking of the UA with HSP90 interaction was conducted using CB-Dock (http://cao.labshare.cn/cb-dock/) [87]. The docking poses were analyzed in both 2D and 3D formats using BIOVIA Discovery Studio 2021, with docking scores obtained from CB-Dock.

### Bioinformatics tools and databases

To identify the targets for UA, SwissTargetPrediction, a ligand-based target prediction web server, was used. The smile code of the UA was obtained from PubChem [88, 89]. The Search Tool database for Retrieval of Interactive Genes (http://string-db.org) utilized to predict potential protein–protein interactions among the candidate genes [90]. The STITCH database (http://stitch.embl.de/) was used to predict protein targets, build a protein-chemical interaction network [91]. Metascape (https://metascape.org) [92] was used to display the network graph results of pathway and process enrichment analysis using UA direct binding protein list. Protein expression data for HSP90AA1 in lung adenocarcinoma and normal lung tissues were obtained from the UALCAN web portal (http://ualcan.path.uab.edu, accessed on 22 June 2025), which provides access to Clinical Proteomic Tumor Analysis Consortium (CPTAC) datasets. Survival analysis, based on The Cancer Genome Atlas (TCGA) data, was also conducted via UALCAN to evaluate the association between HSP90AA1 expression levels and overall survival in lung cancer patients. Pharmacokinetic and toxicity properties of KU and UA were predicted using the pkCSM platform (http://biosig.unimelb.edu.au/pkcsm/) [93].

### Quantification of mRNA expression

RNA was extracted from cells using RNAiso Plus (TaKaRa, Otsu, Japan) following the manufacturer's protocol. Subsequently, 1 μg of RNA was reverse-transcribed into cDNA using M-MLV reverse transcriptase (Invitrogen, Carlsbad, CA, USA). Quantitative analysis of gene expression was performed with SYBR Green (Enzynomics, Seoul, Korea) on a CFX real-time PCR system (Bio-Rad, Hercules, CA, USA). A list of primers can be found in Table S4.

### In vivo antitumor study

Six-week-old male C57BL/6 mice, each weighing between 20 and 22 g, were obtained from Orient Bio (Seongnam, Korea). The animal experiment protocol was approved by the Institutional Animal Care and Use Committee of Sunchon National University (SCNU IACUC-2024–15). All animal handling and in vivo experiments followed the NIH guidelines as outlined in DHEW publication 80–23. The mice were inoculated intratracheally with LLC/iRFP cells (4 × 10^6^ cells in 0.05 mL of PBS per mouse). Five days post-inoculation, the mice were randomly assigned to different groups and administered either the control (distilled water) or KU at doses of 10, 20, or 30 mg/kg/day. Treatment was administered intraperitoneally from day 13 to day 33, with body weight monitored throughout the study. Near-infrared fluorescence bioimaging was performed immediately after treatment using a fluorescence-labeled organism bioimaging instrument (FOBI) system (Cellgentek, Osong, Korea). Upon sacrifice, the lungs were successfully excised, and fluorescence was measured for each sample.

### Flow cytometry analysis with lung tissues

Collagenase D digestion was performed for 30 min at 37 °C after mouse lung tissues were cut into 2 mm fragments. A 70 μm cell stainless steel wire mesh was used to generate a single-cell suspension. The cells were then stained with fluorescence-labeled antibodies. A list of antibodies can be found in Table S5. After 30 min of incubation on ice, the cells will be washed with a FACS buffer. Flow cytometry was performed as a FACS canto II and analyzed with FlowJo 10.4.2 software.

### Immunofluorescences staining

Immunofluorescence staining was performed on lung tumor sections to assess cellular proliferation and HSP90 expression. Tissue sections were fixed in 4% paraformaldehyde, embedded in paraffin. Thereafter, prepared tissues were cut at 3-μm thickness. The following primary antibodies were used: rabbit anti-Ki67 (Abcam, Cat#ab16667, 1:250) and rabbit anti-HSP90 (Cell Signaling Technology, Cat#4877S, 1:100). After incubation, sections were washed three times with PBS and incubated with Alexa Fluor 568-conjugated anti-rabbit IgG secondary antibody (Thermo Fisher, 1:200) for 1 h at room temperature in the dark. Nuclei were counterstained with DAPI for 20 min. After a final wash, sections were mounted with antifade mounting medium and imaged using a fluorescence microscope.

### Statistical analysis

Statistical significance was determined using Student’s t-test with a one-tailed p-value (< 0.05). Cell-based and animal data were analyzed using SigmaPlot 12.5, and ex vivo analyses with GraphPad Prism 5.

## Supplementary Information

Supplementary Material 1

## Data Availability

All data generated or analyzed in this study are available within this published article. The main data supporting the findings of this study are included in the main text and Supplementary Materials.
