# Supplementary material for: Targeting heat shock protein 90 with usnic acid relieves immune suppression via aryl hydrocarbon receptor-mediated mechanisms in lung cancer
Source: Mol Biomed. 2025 Oct 15;6:81. doi: 10.1186/s43556-025-00309-z (PMC12528611; doi:10.1186/s43556-025-00309-z)
Supplement: Supplementary file 1 — Supplementary Material 1 [file 43556_2025_309_MOESM1_ESM.docx]

**Targeting heat shock protein 90 with usnic acid relieves immune suppression via aryl hydrocarbon receptor-mediated mechanisms in lung cancer**

Mücahit Varlı^1^, Eun-Jung Ahn^2^, Suresh R. Bhosle^1^, Kyung-Sub Moon^2^, Hyung-Ho Ha^1^, Hangun Kim^1^,*

^1^College of Pharmacy, Sunchon National University, 255 Jungang-ro, Sunchon, Jeonnam 57922, Republic of Korea; mucahitvarli@s.scnu.ac.kr (M.V.); bhoslesuresh1005@gmail.com (S.R.B.); hhha@sunchon.ac.kr (H.-H.H.)

^2^Department of Neurosurgery, Chonnam National University Hwasun Hospital and Medical School, Hwasun-gun, Jeollanam-do, Republic of Korea

* Correspondence: hangunkim@sunchon.ac.kr; Tel.: +82-61-750-3761


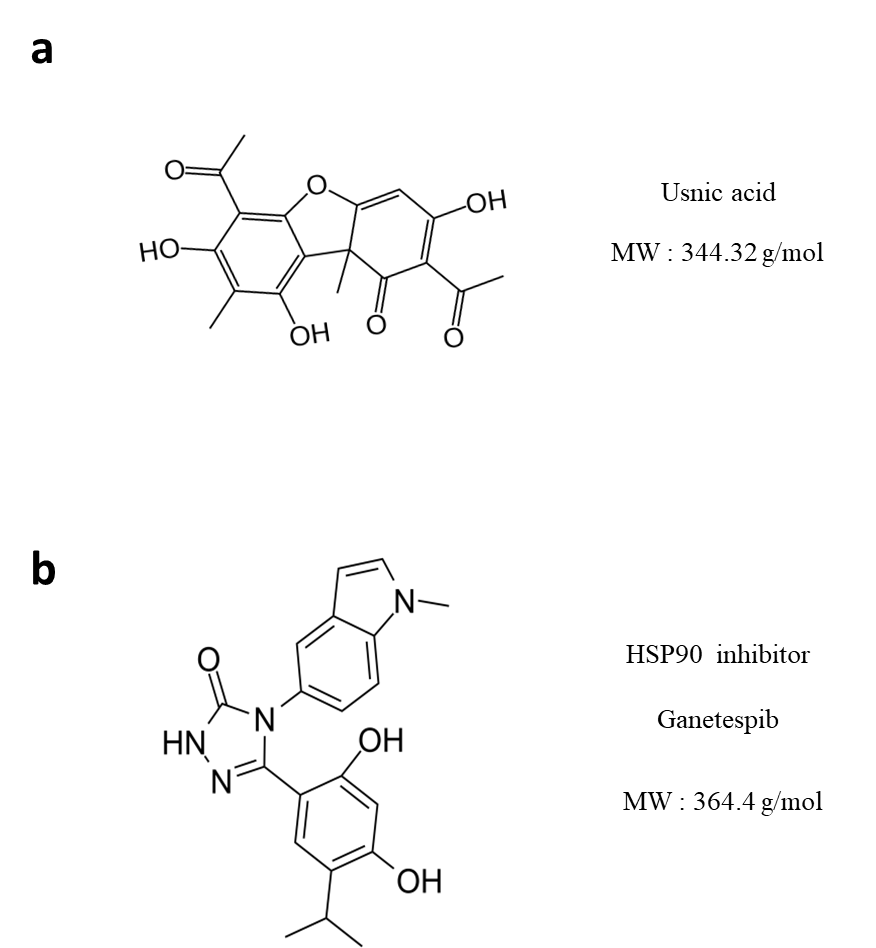


**Fig. S1.** Structure of usnic acid and ganetespib.

**
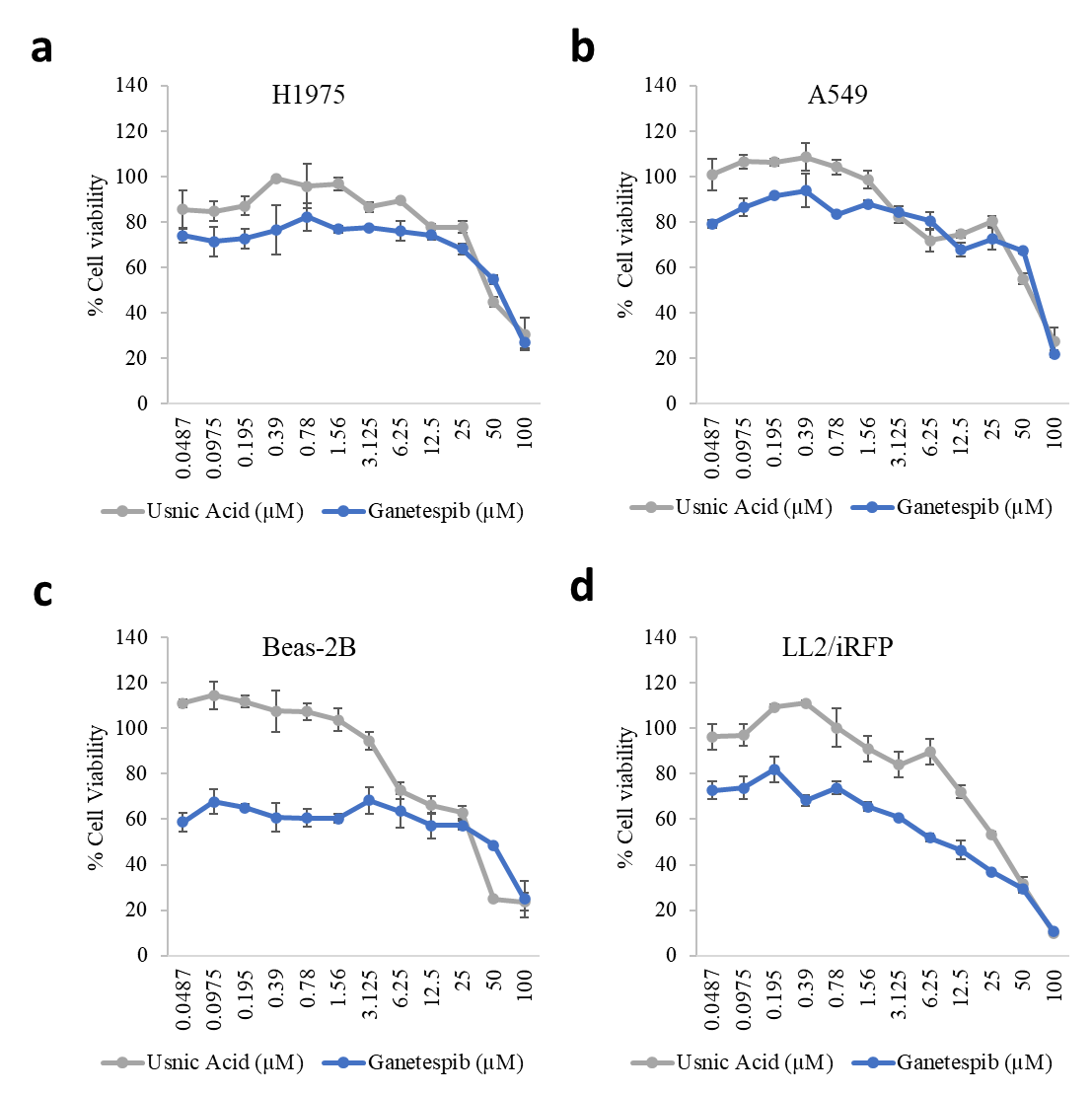
**

**Fig. S2.** Effects of usnic acid and ganetespib on H1975, A549, Beas-2, LLC/iRFP cells. Cells were treated with compounds for 48 hours.

**
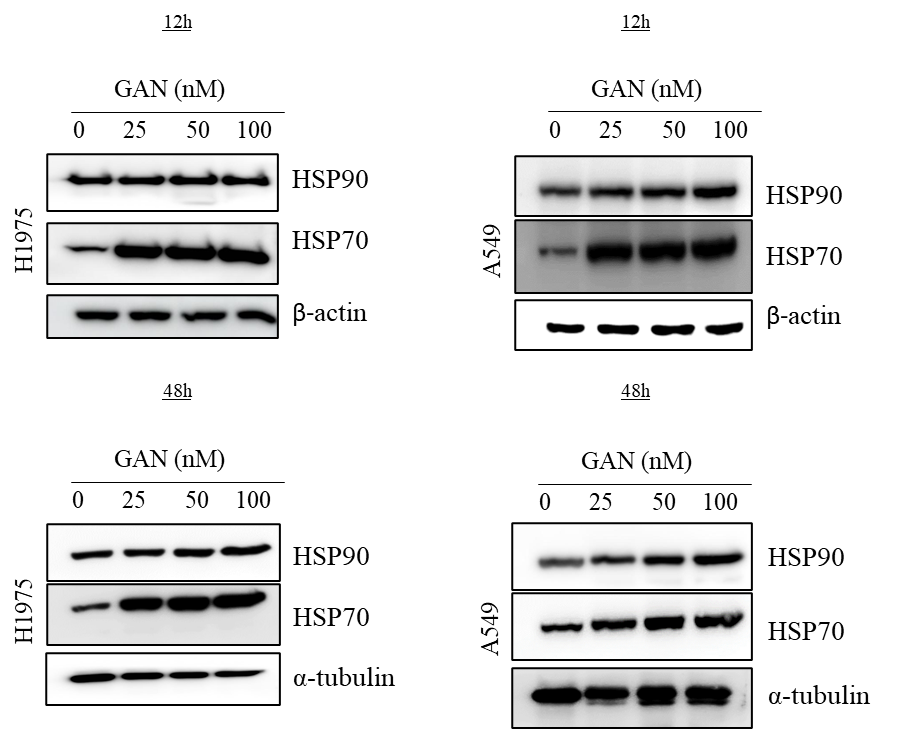
**

**Fig. S3.** Ganetespib upregulated the HSP90 and HSP70 protein level in lung cancer cell line. H1975 and A549 cells were seeded into 6-well plates, cultured for overnight and treated with various concentrations (25, 50, 100 nM) of ganetespib for 12 or 48 h. And then, cell lysates were run on SDS–PAGE gel for subjected to immunoblot assay.

**
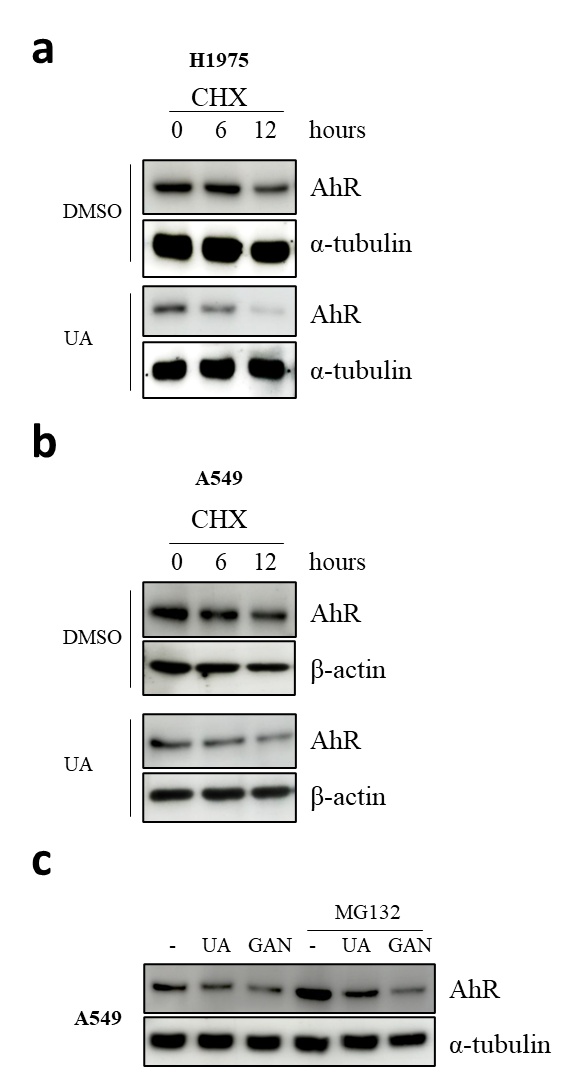
**

**Fig. S4.** (a-b) H1975 and A549 cells treated with DMSO or UA (10 μM) for 24 hours. The protein levels of AhR were determined after treatment with cycloheximide at the indicated times. (c) A549 cells were treated with the proteasome inhibitor MG132 prior to treatment with vehicle control, UA, or GAN and subjected to western blotting to detect AhR protein level. α-tubulin served as the loading control.

**
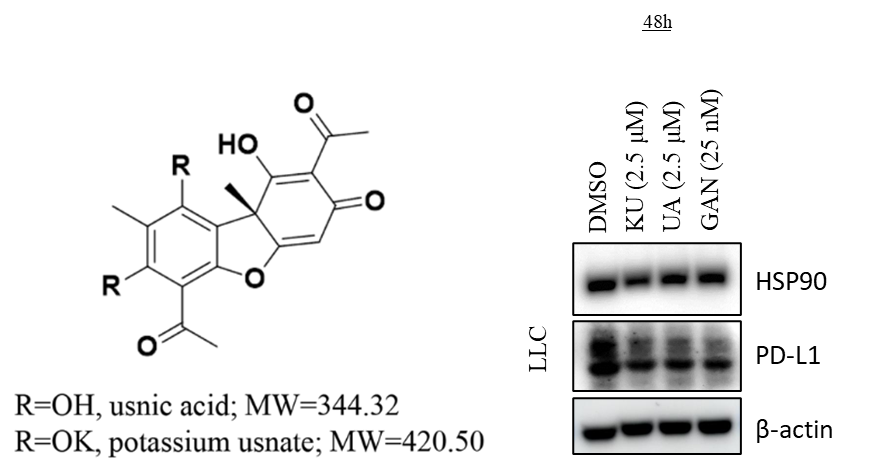
**

**Fig. S5.** (a) Structure of potassium usnate. (b) LLC cells were treated with KU, UA or GAN at indicated concentration for 48 hours. And then, cell lysates were run on SDS–PAGE gel for subjected to immunoblot assay

**Table S1.** **SwissTargetPrediction targeting list.**

| **SwissTargetPrediction** | | | | | | |
| --- | --- | --- | --- | --- | --- | --- |
| **Target** | **Common name** | **Uniprot ID** | **ChEMBL ID** | **Target Class** | **Probability*** | **Known actives (3D/2D)** |
| Prostaglandin E synthase | PTGES | O14684 | CHEMBL5658 | Enzyme | 0.097239989 | 3 / 20 |
| Arachidonate 5-lipoxygenase | ALOX5 | P09917 | CHEMBL215 | Oxidoreductase | 0.097239989 | 2 / 21 |
| Phosphodiesterase 5A | PDE5A | O76074 | CHEMBL1827 | Phosphodiesterase | 0.097239989 | 4 / 0 |
| Egl nine homolog 1 | EGLN1 | Q9GZT9 | CHEMBL5697 | Oxidoreductase | 0.097239989 | 34 / 0 |
| Heat shock protein HSP 90-beta | HSP90AB1 | P08238 | CHEMBL4303 | Other cytosolic protein | 0.097239989 | 18 / 0 |
| Endoplasmin | HSP90B1 | P14625 | CHEMBL1075323 | Other membrane protein | 0.097239989 | 34 / 0 |
| Heat shock protein HSP 90-alpha | HSP90AA1 | P07900 | CHEMBL3880 | Other cytosolic protein | 0.097239989 | 34 / 0 |
| Carbonyl reductase [NADPH] 1 | CBR1 | P16152 | CHEMBL5586 | Enzyme | 0.097239989 | 1 / 0 |
| Serine/threonine-protein kinase mTOR | MTOR | P42345 | CHEMBL2842 | Kinase | 0.097239989 | 1 / 0 |
| PI3-kinase p110-gamma subunit | PIK3CG | P48736 | CHEMBL3267 | Enzyme | 0.097239989 | 6 / 0 |
| PI3-kinase p110-alpha subunit | PIK3CA | P42336 | CHEMBL4005 | Enzyme | 0.097239989 | 9 / 0 |
| Interleukin-8 receptor B | CXCR2 | P25025 | CHEMBL2434 | Family A G protein-coupled receptor | 0.097239989 | 24 / 0 |
| AMP-activated protein kinase, alpha-1 subunit (by homology) | PRKAA1 | Q13131 | CHEMBL4045 | Kinase | 0.097239989 | 1 / 0 |
| Neuropeptide Y receptor type 5 | NPY5R | Q15761 | CHEMBL4561 | Family A G protein-coupled receptor | 0.097239989 | 0 / 21 |
| Peroxisome proliferator-activated receptor gamma | PPARG | P37231 | CHEMBL235 | Nuclear receptor | 0.097239989 | 0 / 17 |
| Apoptosis regulator Bcl-2 | BCL2 | P10415 | CHEMBL4860 | Other ion channel | 0.097239989 | 3 / 0 |
| Tyrosine-protein kinase LCK | LCK | P06239 | CHEMBL258 | Kinase | 0 | 1 / 0 |
| Matrix metalloproteinase 9 | MMP9 | P14780 | CHEMBL321 | Protease | 0 | 2 / 0 |
| Matrix metalloproteinase 1 | MMP1 | P03956 | CHEMBL332 | Protease | 0 | 1 / 0 |
| Matrix metalloproteinase 2 | MMP2 | P08253 | CHEMBL333 | Protease | 0 | 2 / 0 |
| Matrix metalloproteinase 8 | MMP8 | P22894 | CHEMBL4588 | Protease | 0 | 1 / 0 |
| Glucagon receptor | GCGR | P47871 | CHEMBL1985 | Family B G protein-coupled receptor | 0 | 10 / 0 |
| DNA excision repair protein ERCC-5 | ERCC5 | P28715 | CHEMBL4736 | Other nuclear protein | 0 | 2 / 0 |
| Flap endonuclease 1 | FEN1 | P39748 | CHEMBL5027 | Enzyme | 0 | 7 / 0 |
| Calcium-activated potassium channel subunit alpha-1 | KCNMA1 | Q12791 | CHEMBL4304 | Voltage-gated ion channel | 0 | 8 / 0 |
| Cannabinoid receptor 2 | CNR2 | P34972 | CHEMBL253 | Family A G protein-coupled receptor | 0 | 0 / 46 |
| Protein-tyrosine phosphatase 1B | PTPN1 | P18031 | CHEMBL335 | Phosphatase | 0 | 3 / 9 |
| Egl nine homolog 3 | EGLN3 | Q9H6Z9 | CHEMBL5705 | Enzyme | 0 | 8 / 0 |
| Tyrosine-protein kinase receptor FLT3 | FLT3 | P36888 | CHEMBL1974 | Kinase | 0 | 1 / 0 |
| Deoxyribonuclease gamma | DNASE1L3 | Q13609 | CHEMBL1649048 | Enzyme | 0 | 1 / 0 |
| Protein farnesyltransferase | FNTA FNTB | P49354 P49356 | CHEMBL2094108 | Enzyme | 0 | 1 / 0 |
| Nitric oxide synthase, inducible (by homology) | NOS2 | P35228 | CHEMBL4481 | Enzyme | 0 | 4 / 0 |
| Protein kinase C alpha | PRKCA | P17252 | CHEMBL299 | Kinase | 0 | 5 / 1 |
| Protein kinase C beta | PRKCB | P05771 | CHEMBL3045 | Kinase | 0 | 5 / 1 |
| Troponin, cardiac muscle | TNNC1 TNNT2 TNNI3 | P63316 P45379 P19429 | CHEMBL2095202 | Unclassified protein | 0 | 2 / 0 |
| DNA ligase 1 | LIG1 | P18858 | CHEMBL5694 | Enzyme | 0 | 1 / 0 |
| Neurotensin receptor 3 | SORT1 | Q99523 | CHEMBL3091 | Membrane receptor | 0 | 1 / 0 |
| Solute carrier family 22 member 12 | SLC22A12 | Q96S37 | CHEMBL6120 | Electrochemical transporter | 0 | 5 / 0 |
| Dopamine D2 receptor (by homology) | DRD2 | P14416 | CHEMBL217 | Family A G protein-coupled receptor | 0 | 11 / 0 |
| Interleukin-8 receptor A | CXCR1 | P25024 | CHEMBL4029 | Family A G protein-coupled receptor | 0 | 5 / 0 |
| Alkaline phosphatase, tissue-nonspecific isozyme | ALPL | P05186 | CHEMBL5979 | Enzyme | 0 | 1 / 0 |
| Glutamate carboxypeptidase II | FOLH1 | Q04609 | CHEMBL1892 | Protease | 0 | 1 / 0 |
| Heat shock 70 kDa protein 1 | HSPA1A | P0DMV8 | CHEMBL5460 | Other cytosolic protein | 0 | 2 / 0 |
| Dual specificity protein phosphatase 3 | DUSP3 | P51452 | CHEMBL2635 | Phosphatase | 0 | 5 / 0 |
| Protein tyrosine kinase 2 beta | PTK2B | Q14289 | CHEMBL5469 | Kinase | 0 | 1 / 0 |
| Protein kinase C gamma | PRKCG | P05129 | CHEMBL2938 | Kinase | 0 | 4 / 1 |
| Vascular endothelial growth factor A | VEGFA | P15692 | CHEMBL1783 | Secreted protein | 0 | 4 / 0 |
| Heat shock cognate 71 kDa protein | HSPA8 | P11142 | CHEMBL1275223 | Other cytosolic protein | 0 | 1 / 0 |
| Toll-like receptor (TLR7/TLR9) | TLR9 | Q9NR96 | CHEMBL5804 | Toll-like and Il-1 receptors | 0 | 1 / 0 |
| Phosphodiesterase 10A | PDE10A | Q9Y233 | CHEMBL4409 | Phosphodiesterase | 0 | 3 / 0 |
| Glycogen synthase kinase-3 beta | GSK3B | P49841 | CHEMBL262 | Kinase | 0 | 3 / 0 |
| Cyclooxygenase-2 | PTGS2 | P35354 | CHEMBL230 | Oxidoreductase | 0 | 6 / 9 |
| Carbonic anhydrase II | CA2 | P00918 | CHEMBL205 | Lyase | 0 | 10 / 0 |
| Carbonic anhydrase VI | CA6 | P23280 | CHEMBL3025 | Lyase | 0 | 2 / 0 |
| Carbonic anhydrase IV | CA4 | P22748 | CHEMBL3729 | Lyase | 0 | 2 / 0 |
| G-protein coupled receptor 84 | GPR84 | Q9NQS5 | CHEMBL3714079 | Family A G protein-coupled receptor | 0 | 2 / 0 |
| NADPH oxidase 4 | NOX4 | Q9NPH5 | CHEMBL1250375 | Enzyme | 0 | 1 / 0 |
| G-protein coupled receptor 35 | GPR35 | Q9HC97 | CHEMBL1293267 | Family A G protein-coupled receptor | 0 | 2 / 0 |
| Catechol O-methyltransferase | COMT | P21964 | CHEMBL2023 | Transferase | 0 | 14 / 0 |
| Adenosine A2a receptor | ADORA2A | P29274 | CHEMBL251 | Family A G protein-coupled receptor | 0 | 7 / 0 |
| Insulin-like growth factor binding protein 3 | IGFBP3 | P17936 | CHEMBL3997 | Secreted protein | 0 | 1 / 0 |
| Rho-associated protein kinase 2 | ROCK2 | O75116 | CHEMBL2973 | Kinase | 0 | 1 / 0 |
| G protein-coupled receptor kinase 6 | GRK6 | P43250 | CHEMBL6144 | Kinase | 0 | 2 / 0 |
| Integrin alpha-V/beta-3 | ITGAV ITGB3 | P06756 P05106 | CHEMBL1907598 | Membrane receptor | 0 | 4 / 0 |
| ALK tyrosine kinase receptor | ALK | Q9UM73 | CHEMBL4247 | Kinase | 0 | 2 / 0 |
| Lysine-specific demethylase 4C | KDM4C | Q9H3R0 | CHEMBL6175 | Eraser | 0 | 1 / 0 |
| Carbonic anhydrase I | CA1 | P00915 | CHEMBL261 | Lyase | 0 | 8 / 0 |
| Carbonic anhydrase XII | CA12 | O43570 | CHEMBL3242 | Lyase | 0 | 8 / 0 |
| Protein kinase C epsilon | PRKCE | Q02156 | CHEMBL3582 | Kinase | 0 | 4 / 0 |
| Carbonic anhydrase IX | CA9 | Q16790 | CHEMBL3594 | Lyase | 0 | 8 / 0 |
| GABA-A receptor; alpha-3/beta-3/gamma-2 | GABRB3 GABRA3 GABRG2 | P28472 P34903 P18507 | CHEMBL2094120 | Ligand-gated ion channel | 0 | 2 / 0 |
| GABA-A receptor; alpha-1/beta-3/gamma-2 | GABRB3 GABRG2 GABRA1 | P28472 P18507 P14867 | CHEMBL2094121 | Ligand-gated ion channel | 0 | 2 / 0 |
| Mannose-6-phosphate isomerase | MPI | P34949 | CHEMBL2758 | Isomerase | 0 | 1 / 0 |
| Histone deacetylase 1 | HDAC1 | Q13547 | CHEMBL325 | Eraser | 0 | 3 / 0 |
| Dihydroorotate dehydrogenase (by homology) | DHODH | Q02127 | CHEMBL1966 | Oxidoreductase | 0 | 8 / 0 |
| Thyroid hormone receptor alpha | THRA | P10827 | CHEMBL1860 | Nuclear receptor | 0 | 1 / 0 |
| Thyroid hormone receptor beta-1 | THRB | P10828 | CHEMBL1947 | Nuclear receptor | 0 | 1 / 0 |
| Adenosine A1 receptor (by homology) | ADORA1 | P30542 | CHEMBL226 | Family A G protein-coupled receptor | 0 | 2 / 0 |
| MAP kinase signal-integrating kinase 2 | MKNK2 | Q9HBH9 | CHEMBL4204 | Kinase | 0 | 1 / 1 |
| MAP kinase-interacting serine/threonine-protein kinase MNK1 | MKNK1 | Q9BUB5 | CHEMBL4718 | Kinase | 0 | 1 / 1 |
| Serine/threonine-protein kinase/endoribonuclease IRE1 | ERN1 | O75460 | CHEMBL1163101 | Enzyme | 0 | 8 / 0 |
| Equilibrative nucleoside transporter 1 | SLC29A1 | Q99808 | CHEMBL1997 | Electrochemical transporter | 0 | 2 / 0 |
| CCR4-NOT transcription complex subunit 7 | CNOT7 | Q9UIV1 | CHEMBL3616361 | Hydrolase | 0 | 3 / 0 |
| Glyoxalase I | GLO1 | Q04760 | CHEMBL2424 | Enzyme | 0 | 2 / 0 |
| Estradiol 17-beta-dehydrogenase 1 | HSD17B1 | P14061 | CHEMBL3181 | Enzyme | 0 | 4 / 0 |
| Thrombopoietin receptor | MPL | P40238 | CHEMBL1864 | Membrane receptor | 0 | 6 / 0 |
| Adenosine A2b receptor | ADORA2B | P29275 | CHEMBL255 | Family A G protein-coupled receptor | 0 | 1 / 0 |
| Adenosine A3 receptor | ADORA3 | P0DMS8 | CHEMBL256 | Family A G protein-coupled receptor | 0 | 1 / 0 |
| Serine/threonine-protein kinase RAF | RAF1 | P04049 | CHEMBL1906 | Kinase | 0 | 1 / 0 |
| C-C chemokine receptor type 4 | CCR4 | P51679 | CHEMBL2414 | Family A G protein-coupled receptor | 0 | 2 / 0 |
| Inhibitor of nuclear factor kappa B kinase beta subunit | IKBKB | O14920 | CHEMBL1991 | Kinase | 0 | 5 / 4 |
| Proteasome assembly chaperone 3 | PSMG3 | Q9BT73 | CHEMBL1075137 | Unclassified protein | 0 | 1 / 0 |
| Protein-tyrosine phosphatase 2C | PTPN11 | Q06124 | CHEMBL3864 | Phosphatase | 0 | 1 / 0 |
| Cytochrome P450 19A1 | CYP19A1 | P11511 | CHEMBL1978 | Cytochrome P450 | 0 | 0 / 18 |
| Dual specificity mitogen-activated protein kinase kinase 1 | MAP2K1 | Q02750 | CHEMBL3587 | Kinase | 0 | 1 / 0 |
| Sodium channel protein type IX alpha subunit | SCN9A | Q15858 | CHEMBL4296 | Voltage-gated ion channel | 0 | 1 / 0 |
| NADH-ubiquinone oxidoreductase chain 4 | MT-ND4 | P03905 | CHEMBL4499 | Oxidoreductase | 0 | 0 / 4 |
| Phosphodiesterase 4D | PDE4D | Q08499 | CHEMBL288 | Phosphodiesterase | 0 | 2 / 0 |
| Tyrosine-protein kinase BRK | PTK6 | Q13882 | CHEMBL4601 | Kinase | 0 | 3 / 0 |
| Cyclin-dependent kinase 4/cyclin D1 | CCND1 CDK4 | P24385 P11802 | CHEMBL1907601 | Kinase | 0 | 2 / 0 |

**Table S2. Usnic acid binding list with chemical prob.**

| **Accession** | **Coverage** | **# PSMs** | **# Peptides** | **# AAs** | **MW [kDa]** | **calc. pI** | **Score** | **Description** |
| --- | --- | --- | --- | --- | --- | --- | --- | --- |
| P04264 | 20.81 | 17 | 10 | 644 | 66.0 | 8.12 | 80.39 | Keratin, type II cytoskeletal 1 OS=Homo sapiens GN=KRT1 PE=1 SV=6 - [K2C1_HUMAN] |
| P35527 | 34.51 | 13 | 9 | 623 | 62.0 | 5.24 | 73.74 | Keratin, type I cytoskeletal 9 OS=Homo sapiens GN=KRT9 PE=1 SV=3 - [K1C9_HUMAN] |
| P13645 | 18.15 | 10 | 8 | 584 | 58.8 | 5.21 | 58.22 | Keratin, type I cytoskeletal 10 OS=Homo sapiens GN=KRT10 PE=1 SV=6 - [K1C10_HUMAN] |
| P13639 | 14.34 | 11 | 7 | 858 | 95.3 | 6.83 | 44.19 | Elongation factor 2 OS=Homo sapiens GN=EEF2 PE=1 SV=4 - [EF2_HUMAN] |
| P35908 | 10.80 | 8 | 6 | 639 | 65.4 | 8.00 | 35.46 | Keratin, type II cytoskeletal 2 epidermal OS=Homo sapiens GN=KRT2 PE=1 SV=2 - [K22E_HUMAN] |
| Q9BQE3 | 19.15 | 6 | 6 | 449 | 49.9 | 5.10 | 24.59 | Tubulin alpha-1C chain OS=Homo sapiens GN=TUBA1C PE=1 SV=1 - [TBA1C_HUMAN] |
| P60709 | 12.53 | 5 | 4 | 375 | 41.7 | 5.48 | 24.49 | Actin, cytoplasmic 1 OS=Homo sapiens GN=ACTB PE=1 SV=1 - [ACTB_HUMAN] |
| P07900 | 8.88 | 4 | 4 | 732 | 84.6 | 5.02 | 24.43 | Heat shock protein HSP 90-alpha OS=Homo sapiens GN=HSP90AA1 PE=1 SV=5 - [HS90A_HUMAN] |
| P68104 | 8.23 | 3 | 3 | 462 | 50.1 | 9.01 | 20.81 | Elongation factor 1-alpha 1 OS=Homo sapiens GN=EEF1A1 PE=1 SV=1 - [EF1A1_HUMAN] |
| P08107 | 8.42 | 2 | 2 | 641 | 70.0 | 5.66 | 19.99 | Heat shock 70 kDa protein 1A/1B OS=Homo sapiens GN=HSPA1A PE=1 SV=5 - [HSP71_HUMAN] |
| P11142 | 10.22 | 3 | 3 | 646 | 70.9 | 5.52 | 19.54 | Heat shock cognate 71 kDa protein OS=Homo sapiens GN=HSPA8 PE=1 SV=1 - [HSP7C_HUMAN] |
| P08238 | 5.39 | 3 | 3 | 724 | 83.2 | 5.03 | 19.25 | Heat shock protein HSP 90-beta OS=Homo sapiens GN=HSP90AB1 PE=1 SV=4 - [HS90B_HUMAN] |
| P07437 | 12.61 | 3 | 3 | 444 | 49.6 | 4.89 | 16.80 | Tubulin beta chain OS=Homo sapiens GN=TUBB PE=1 SV=2 - [TBB5_HUMAN] |
| P10809 | 12.57 | 5 | 5 | 573 | 61.0 | 5.87 | 16.37 | 60 kDa heat shock protein, mitochondrial OS=Homo sapiens GN=HSPD1 PE=1 SV=2 - [CH60_HUMAN] |
| Q9HBK9 | 12.80 | 3 | 2 | 375 | 41.7 | 6.20 | 15.49 | Arsenite methyltransferase OS=Homo sapiens GN=AS3MT PE=1 SV=3 - [AS3MT_HUMAN] |
| Q06830 | 10.55 | 2 | 2 | 199 | 22.1 | 8.13 | 14.59 | Peroxiredoxin-1 OS=Homo sapiens GN=PRDX1 PE=1 SV=1 - [PRDX1_HUMAN] |
| P06733 | 8.99 | 2 | 2 | 434 | 47.1 | 7.39 | 13.94 | Alpha-enolase OS=Homo sapiens GN=ENO1 PE=1 SV=2 - [ENOA_HUMAN] |
| P07195 | 9.28 | 2 | 2 | 334 | 36.6 | 6.05 | 13.88 | L-lactate dehydrogenase B chain OS=Homo sapiens GN=LDHB PE=1 SV=2 - [LDHB_HUMAN] |
| P04259 | 5.50 | 3 | 3 | 564 | 60.0 | 8.00 | 12.88 | Keratin, type II cytoskeletal 6B OS=Homo sapiens GN=KRT6B PE=1 SV=5 - [K2C6B_HUMAN] |
| P02768 | 2.46 | 2 | 1 | 609 | 69.3 | 6.28 | 12.16 | Serum albumin OS=Homo sapiens GN=ALB PE=1 SV=2 - [ALBU_HUMAN] |
| P04406 | 12.84 | 3 | 3 | 335 | 36.0 | 8.46 | 11.16 | Glyceraldehyde-3-phosphate dehydrogenase OS=Homo sapiens GN=GAPDH PE=1 SV=3 - [G3P_HUMAN] |
| P00338 | 7.23 | 2 | 2 | 332 | 36.7 | 8.27 | 10.99 | L-lactate dehydrogenase A chain OS=Homo sapiens GN=LDHA PE=1 SV=2 - [LDHA_HUMAN] |
| P23528 | 25.30 | 2 | 2 | 166 | 18.5 | 8.09 | 10.44 | Cofilin-1 OS=Homo sapiens GN=CFL1 PE=1 SV=3 - [COF1_HUMAN] |
| P62258 | 10.98 | 2 | 2 | 255 | 29.2 | 4.74 | 10.23 | 14-3-3 protein epsilon OS=Homo sapiens GN=YWHAE PE=1 SV=1 - [1433E_HUMAN] |
| P14618 | 8.29 | 3 | 3 | 531 | 57.9 | 7.84 | 9.98 | Pyruvate kinase isozymes M1/M2 OS=Homo sapiens GN=PKM PE=1 SV=4 - [KPYM_HUMAN] |
| P63241 | 15.58 | 2 | 2 | 154 | 16.8 | 5.24 | 9.23 | Eukaryotic translation initiation factor 5A-1 OS=Homo sapiens GN=EIF5A PE=1 SV=2 - [IF5A1_HUMAN] |
| P60842 | 4.19 | 1 | 1 | 406 | 46.1 | 5.48 | 8.70 | Eukaryotic initiation factor 4A-I OS=Homo sapiens GN=EIF4A1 PE=1 SV=1 - [IF4A1_HUMAN] |
| P27348 | 5.71 | 1 | 1 | 245 | 27.7 | 4.78 | 6.98 | 14-3-3 protein theta OS=Homo sapiens GN=YWHAQ PE=1 SV=1 - [1433T_HUMAN] |
| P62937 | 5.45 | 1 | 1 | 165 | 18.0 | 7.81 | 6.60 | Peptidyl-prolyl cis-trans isomerase A OS=Homo sapiens GN=PPIA PE=1 SV=2 - [PPIA_HUMAN] |
| P04075 | 6.04 | 1 | 1 | 364 | 39.4 | 8.09 | 6.56 | Fructose-bisphosphate aldolase A OS=Homo sapiens GN=ALDOA PE=1 SV=2 - [ALDOA_HUMAN] |
| P63104 | 8.16 | 1 | 1 | 245 | 27.7 | 4.79 | 6.54 | 14-3-3 protein zeta/delta OS=Homo sapiens GN=YWHAZ PE=1 SV=1 - [1433Z_HUMAN] |
| P02787 | 5.44 | 1 | 1 | 698 | 77.0 | 7.12 | 5.59 | Serotransferrin OS=Homo sapiens GN=TF PE=1 SV=3 - [TRFE_HUMAN] |
| P62826 | 5.09 | 1 | 1 | 216 | 24.4 | 7.49 | 4.79 | GTP-binding nuclear protein Ran OS=Homo sapiens GN=RAN PE=1 SV=3 - [RAN_HUMAN] |
| P25705 | 3.98 | 1 | 1 | 553 | 59.7 | 9.13 | 4.74 | ATP synthase subunit alpha, mitochondrial OS=Homo sapiens GN=ATP5A1 PE=1 SV=1 - [ATPA_HUMAN] |
| P81605 | 10.00 | 1 | 1 | 110 | 11.3 | 6.54 | 4.54 | Dermcidin OS=Homo sapiens GN=DCD PE=1 SV=2 - [DCD_HUMAN] |
| P19338 | 3.10 | 1 | 1 | 710 | 76.6 | 4.70 | 4.29 | Nucleolin OS=Homo sapiens GN=NCL PE=1 SV=3 - [NUCL_HUMAN] |
| P22314 | 2.17 | 1 | 1 | 1058 | 117.8 | 5.76 | 4.19 | Ubiquitin-like modifier-activating enzyme 1 OS=Homo sapiens GN=UBA1 PE=1 SV=3 - [UBA1_HUMAN] |
| Q07021 | 10.64 | 1 | 1 | 282 | 31.3 | 4.84 | 4.16 | Complement component 1 Q subcomponent-binding protein, mitochondrial OS=Homo sapiens GN=C1QBP PE=1 SV=1 - [C1QBP_HUMAN] |
| Q32P51 | 5.00 | 1 | 1 | 320 | 34.2 | 9.00 | 4.13 | Heterogeneous nuclear ribonucleoprotein A1-like 2 OS=Homo sapiens GN=HNRNPA1L2 PE=2 SV=2 - [RA1L2_HUMAN] |
| P12277 | 4.20 | 1 | 1 | 381 | 42.6 | 5.59 | 3.97 | Creatine kinase B-type OS=Homo sapiens GN=CKB PE=1 SV=1 - [KCRB_HUMAN] |
| P24534 | 6.67 | 1 | 1 | 225 | 24.7 | 4.67 | 3.96 | Elongation factor 1-beta OS=Homo sapiens GN=EEF1B2 PE=1 SV=3 - [EF1B_HUMAN] |
| P22626 | 4.53 | 1 | 1 | 353 | 37.4 | 8.95 | 3.93 | Heterogeneous nuclear ribonucleoproteins A2/B1 OS=Homo sapiens GN=HNRNPA2B1 PE=1 SV=2 - [ROA2_HUMAN] |
| P07737 | 11.43 | 1 | 1 | 140 | 15.0 | 8.27 | 3.52 | Profilin-1 OS=Homo sapiens GN=PFN1 PE=1 SV=2 - [PROF1_HUMAN] |
| P10599 | 12.38 | 1 | 1 | 105 | 11.7 | 4.92 | 3.46 | Thioredoxin OS=Homo sapiens GN=TXN PE=1 SV=3 - [THIO_HUMAN] |
| P48643 | 1.85 | 1 | 1 | 541 | 59.6 | 5.66 | 3.07 | T-complex protein 1 subunit epsilon OS=Homo sapiens GN=CCT5 PE=1 SV=1 - [TCPE_HUMAN] |
| P13797 | 1.90 | 1 | 1 | 630 | 70.8 | 5.60 | 3.02 | Plastin-3 OS=Homo sapiens GN=PLS3 PE=1 SV=4 - [PLST_HUMAN] |
| P22492 | 5.31 | 1 | 1 | 207 | 22.0 | 11.71 | 2.79 | Histone H1t OS=Homo sapiens GN=HIST1H1T PE=2 SV=4 - [H1T_HUMAN] |
| P00558 | 2.16 | 1 | 1 | 417 | 44.6 | 8.10 | 2.69 | Phosphoglycerate kinase 1 OS=Homo sapiens GN=PGK1 PE=1 SV=3 - [PGK1_HUMAN] |
| Q13206B18A8:  B50I25A27:  B50A2:  B50I25A27:  B50AA2:A50 | 0.80 | 1 | 1 | 875 | 100.8 | 8.63 | 1.61 | Probable ATP-dependent RNA helicase DDX10 OS=Homo sapiens GN=DDX10 PE=1 SV=2 - [DDX10_HUMAN] |

**Table S3. Antibody information**

| Antibody name | Product Information | Application | Dilution |
| --- | --- | --- | --- |
| PD-L1 (E1L3N) XP Rabbit mAb | Cell signaling, #13684 | Flow cytometry | 1:500 |
|  |  | Western blotting | 1:1000 |
| CD275 (B7-H2) Monoclonal Antibody (MIH12), Functional Grade, eBioscience™ | Invitrogen, #16-5859-82 | Flow cytometry | 1:1000 |
| Anti-rabbit IgG (H+L), F(ab')2 Fragment | Cell signaling Alexa Fluor® 488 Conjugate, #4412 | Flow cytometry | 1:1000 |
| AhR (D5S6H) Rabbit mAb | Cell signaling, #83200 | Western blotting | 1:1000 |
| α-Tubulin (11H10) Rabbit mAB | Cell signaling, #2125 | Western blotting | 1:1000 |
| Anti-Lamin B1 Antibody – Nuclear Envelope Marker | Abcam, ab16048 | Western blotting | 1:1000 |
| HSP70 (6B3) Rat mAB | Cell signaling, #4873 | Western blotting | 1:1000 |
| HSP90 (C45G5) Rabbit mAB | Cell signaling, #4877 | Western blotting | 1:1000 |
| Goat Anti-Rabbit IgG (H+L) Peroxidase Conjugated | Thermo scientific, 31460 | Western blotting | 1:5000 |
| Goat Anti-Mouse IgG (H+L) Peroxidase Conjugated | Thermo  scientific, NCI1430KR | Western blotting | 1:5000 |
| Goat Anti-Rat IgG (H+L) Peroxidase Conjugated | Thermo scientific, 31470 | Western blotting | 1:5000 |

**Table S4. Primer sequences for qRT-PCR.**

| Gene symbol | Primer sequences | |
| --- | --- | --- |
|  | For (5'-3') | Rev (5'-3') |
| *H_ARNT* | TGTGGACCCAGTTTCTGTGA | GACCACCACGAAGTGAGGTT |
| *H_ICOSL* | AGCGTTGAGGTTACACTGCATGTGGC | GCTGACCACGTCATACAAGCCCCGCA |
| *H_PD-L1* | GGAGATTAGATCCTGAGGAAAACCA | AACGGAAGATGAATGTCAGTGCTA |
| *H_AHR* | ATTGTGCCGAGTCCCATATC | AAGCAGGCGTGCATTAGACT |
| *H_AFMID* | AGCGGTATCCAAGCAACAAGG | AGACCCCACTCACCAGGAAAA |
| *H_AHRR* | AGCGGAGATGAAAATGAGGA | AGTTCCGATTCGCACAGACT |
| *H_CYP1A1* | GATTGAGCACTGTCAGGAGAAGC | ATGAGGCTCCAGGAGATAGCAG |
| *H_CYP1B1* | GACGCCTTTATCCTCTCTGCG | ACGACCTGATCCAATTCTGCC |
| *H_IDO1* | GATCATCTCACAGACCACAAGTCACAG | CTTGGAGAGTTGGCAGTAAGGAACAG |
| *H_SLC1A5* | AGAATGTACTTGCCAAGGCG | CACCATGGTTCTGGTCTCCT |
| *H_SLC7A7* | CTCACTGCTTAACGGCGTGT | CCAGTTCCGCATAACAAAGG |
| *H_GAPDH* | GAAGGTGAAGGTCGGAGTC | GAAGATGGTGATGGGATTTC |
| M_Slc7a5 | GGTCTCTGTTCACGTCCTCAAG | GAACACCAGTGATGGCACAGGT |
| M_Slc1a5 | CTGCCTGTGAAGGACATCTCCT | CTCGGCATCTTGGTTCGATCCA |
| M_Ido1 | GCAGACTGTGTCCTGGCAAACT | AGAGACGAGGAAGAAGCCCTTG |
| M_Afmid | CTTTCTCCTGGTGAGTGGGATC | CTGGAACCACATCCAAGTGTCG |
| M_Pd-l1 | TGCGGACTACAAGCGAATCACG | CTCAGCTTCTGGATAACCCTCG |
| M_Icosl | CAGCGGCATTCGTTTCCTTC | GTCAGGCGTGGTCTGTAAGT |
| M_β-actin | ATTGTGAACTTTGGGGGATG | GATGAGATTGGCATGGCTTT |

| **Target** | **Fluorochrome** | **Manufacturer** |
| --- | --- | --- |
| CD45 | FITC | eBioscience |
| CD11b | PE | eBioscience |
| CD4 | APC | eBioscience |
| CD8 | APC | eBioscience |
| CD279 (PD-1) | PE | eBioscience |

**Table S5. Antibody information for ex vivo FACS analysis**
